# Effective behavior change techniques to promote physical activity in adults with overweight or obesity: A systematic review and meta‐analysis

**DOI:** 10.1111/obr.13258

**Published:** 2021-05-05

**Authors:** Eliana Carraça, Jorge Encantado, Francesca Battista, Kristine Beaulieu, John Blundell, Luca Busetto, Marleen van Baak, Dror Dicker, Andrea Ermolao, Nathalie Farpour‐Lambert, Adriyan Pramono, Euan Woodward, Alice Bellicha, Jean‐Michel Oppert

**Affiliations:** ^1^ CIDEFES Universidade Lusófona de Humanidades e Tecnologias, Faculdade de Educação Física e Desporto Lisbon Portugal; ^2^ Applied Psychology Research Center Capabilities & Inclusion ISPA – University Institute Lisbon Portugal; ^3^ Sport and Exercise Medicine Division, Department of Medicine University of Padova Padova Italy; ^4^ Appetite Control and Energy Balance Group, School of Psychology, Faculty of Medicine and Health University of Leeds Leeds UK; ^5^ Obesity Management Task Force European Association for the Study of Obesity Teddington UK; ^6^ Department of Medicine University of Padova Padova Italy; ^7^ NUTRIM School of Nutrition and Translational Research in Metabolism, Department of Human Biology Maastricht University Medical Centre+ Maastricht The Netherlands; ^8^ Department of Internal Medicine D Hasharon Hospital, Rabin Medical Center Petah Tikva Israel; ^9^ Obesity Prevention and Care Program Contrepoids, Service of Endocrinology, Diabetology, Nutrition and Therapeutic Patient Education, Department of Internal Medicine University Hospitals of Geneva and University of Geneva Geneva Switzerland; ^10^ INSERM, Nutrition and Obesities: Systemic Approaches, NutriOmics Sorbonne University Paris France; ^11^ UFR SESS‐STAPS University Paris‐Est Créteil Créteil France; ^12^ Assistance Publique‐Hôpitaux de Paris (AP‐HP), Pitié‐Salpêtrière Hospital, Department of Nutrition, Institute of Cardiometabolism and Nutrition Sorbonne University Paris France

**Keywords:** BCTs, obesity, overweight, physical activity

## Abstract

Multicomponent behavior change interventions are typically used in weight management, but results are largely heterogeneous and modest. Determining which techniques (behavior change technique [BCTs]) are more effective in changing behavior is thus required. This study aimed to identify the most effective BCTs for increasing physical activity (PA) in digital and face‐to‐face behavior change interventions in adults with overweight/obesity. Four databases were searched for eligible studies until October 2019. BCTs were coded using BCTTv1 and MBCT taxonomies. Sixty‐two RCTs were included. Meta‐regressions were performed to explore BCTs' moderating role. Five BCTs showed significant moderator effects on PA in digital interventions: *goal setting behavior*, *goal setting outcome*, *graded tasks*, *social incentive*, and *self‐monitoring of behavior* (adjusted *R* ^2^'s = 0.15–0.51). One BCT showed significant moderator effects on PA in face‐to‐face interventions, *behavioral practice and rehearsal* (adjusted *R* ^2^ = 0.22). Multivariate and sensitivity analysis generally led to similar findings. Effective BCTs for increasing PA in adults with overweight/obesity in digital and face‐to‐face interventions seem to differ. Evidence suggests that using *goal setting*, *social incentive*, and *graded tasks* might help improve PA in digital interventions while avoiding inconsistent *self‐monitoring of behavior*. In face‐to‐face interventions, prompting *behavioral practice and rehearsal* might lead to better PA outcomes. Still, further studies are needed. Implications of the current findings are discussed.

## 1 INTRODUCTION

Obesity prevalence increased to pandemic proportions from 1975 to 2016, now reaching approximately 20% worldwide.^1^ This constitutes a major health and social problem. Behavioral interventions targeting mainly changes in diet and physical activity (PA) are the cornerstone of nonpharmacological interventions for weight management, in populations with overweight and obesity.^2^ According to previous reviews, interventions designed to change weight‐related behaviors generally include many components and typically produce small effects, though with considerable heterogeneity in effectiveness.^3^, ^4^ This calls for further investigation into the identification of the most effective intervention components (i.e., behavior change techniques [BCTs]) in promoting successful weight management and related behaviors like PA.^5^

The development of comprehensive taxonomies of intervention techniques, commonly referred to as BCTs,^6^ has largely contributed to improvements in the identification, comparison, and reporting of intervention content through a systematic examination of intervention components. Prior systematic reviews^7^, ^8^ have confirmed the feasibility of using this taxonomy for thorough methodical analyses of behavior change interventions. Still, this taxonomy is not exhaustive, as it leaves out BCTs related to the interpersonal motivational climate. To overcome this shortcoming, a new taxonomy on motivational behavior change techniques (MBCTs) was produced very recently.^9^ The use of both these taxonomies as the base for coding and identifying the active ingredients (BCTs) in behavioral interventions will allow for the examination of those that are more likely to lead to more successful/favorable outcomes in a more comprehensive way and thus allow to design more effective interventions and improve the use of limited resources.^10^

Prior reviews have already attempted to identify the most effective BCTs in promoting PA in adults with overweight/obesity. Some could not find consistent and sufficient evidence to accurately understand how theory‐based interventions succeeded or failed to change PA in adults with overweight/obesity.^11^ Others did not find any effective BCTs for changing PA behavior in PA‐only or multi‐behavior interventions (≥12 weeks) in adults with obesity and related comorbidities or risk factors, nor associations between the number of BCTs and PA changes.^12^ The latter findings suggested that the quality and combination of specific BCTs rather than the quantity were more likely to affect the outcomes. Nevertheless, a few BCTS were identified as potentially useful to increase PA, namely, *provision of instructions*, *self‐monitoring*, *relapse prevention*, and *prompting practice*.^12^ Another review found that 21 BCTs were associated with positive changes in PA in multi‐behavior change interventions for adults with obesity (BMI > 30 kg/m^2^) using especially *teach‐to‐use prompts/cues*, *prompt practice*, or *prompt rewards contingent on effort or progress toward behavior*.^13^ More recently, Samdal et al.^8^ found that the number of BCTs unique to the intervention group and the BCTs' *goal setting* and *self‐monitoring of behavior* predicted short‐ and long‐term PA changes in multi‐behavior change interventions (≥12 weeks) for adults with overweight/obesity.

Using set combinations of theory‐congruent techniques has also been suggested to result in more favorable outcomes in the general population. Michie et al.^7^ found that interventions combining self‐monitoring with one or more BCTs derived from control theory (e.g., goal setting and provision of feedback) were more effective in promoting changes in PA and healthy eating than interventions not using these techniques. Similar effects were found in other meta‐analyses, including populations with obesity.^12^ In addition, a recent systematic review of reviews suggested that interventions based on control theory, self‐determination theory, and motivational interviewing were apparently more successful in changing PA.^14^

Digital behavior change interventions (i.e., involving the use of computer technology or digital encoding of information)^10^ are a viable option for weight management as they have the potential for wide reach at low cost and can be adapted to individual needs and the information can be delivered in an engaging and interactive way, with greater fidelity to intervention content.^10^, ^15^ Although digital interventions are promising for health behavior change, research on their effects is still in an early stage. In populations with overweight/obesity, previous reviews reported positive but often small effects with considerable variability^16^ and revealed that interventions providing stress management and communication skills (used in few interventions), and using a greater number of BCTs, had larger effects on health‐related behaviors.^17^

Despite the impressive scope of these meta‐analytic reviews, they have shortcomings. Previous reviews have either failed to analyze BCT effectiveness,^18^, ^19^ or have analyzed BCTs using less comprehensive taxonomies, leaving out motivational techniques,^8^, ^12^, ^13^ and none has separately analyzed digital and face‐to face interventions effect on PA. In addition, most reviews used meta‐analysis and/or univariate regression rather than multivariate meta‐regressions, and the only study using multivariate analysis mixed intervention effects on PA and diet.^8^ Finally, most reviews did not analyze the moderating role of the type of PA measure used (objective vs. self‐report), which can lead to distorted findings in analytic models due to the inaccurate PA estimation observed in higher BMI categories self‐reporting.^20^, ^21^

To address these shortcomings, a working group of European clinical and nonclinical obesity experts was convened in 2019 under the auspices of the European Association for the Study of Obesity (EASO). Hence, in the context of the EASO Physical Activity (PA) Working Group, this systematic review and meta‐analysis focused on one key question: What are the most effective BCTs, or theory‐driven combinations of BCTs, for increasing PA in behavior change interventions targeting adults with overweight or obesity, delivered in digital and in face‐to‐face format? Accordingly, this study had three specific goals: (i) examine which BCTs were most frequently used in behavior change interventions (in digital and face‐to‐face format); (ii) identify which BCTs were unique or mainly used in each intervention format; and (iii) examine which BCTs, or possible theory‐driven combinations, were most effective in each intervention format. If the available evidence allows, this will contribute to the development of recommendations for the design of effective interventions to promote PA in adults with overweight/obesity.

## 2 METHODS

This systematic review follows the Preferred Reporting Items for Systematic Reviews and Meta‐Analysis (PRISMA) guidelines^22^ and is registered in the PROSPERO database (registration number CRD42019157823).

### 2.1 Search strategy

Four electronic databases (PubMed, Web of Science, PsycInfo, and SportDiscus) were searched for original articles published in English up to October 2019 (including online ahead of print publication) using a comprehensive search strategy, combining terms concerning the population of interest, the evaluated exposure(s), the primary outcome of this review, and study design. A full search example can be found in Table S1. Previous systematic reviews were screened to identify relevant subject headings and key words to include within each subject category. Reference lists from the resulting reviews and articles were also screened to identify additional articles.

### 2.2 Study selection, inclusion, and exclusion

Articles were included if they were published in English in peer‐reviewed journals and included adult‐only samples (≥ 18 years including older adults) with overweight (BMI ≥ 25 kg/m^2^) or obesity (BMI ≥ 30 kg/m^2^) participating in interventions (primarily or secondarily) aimed at increasing PA. Randomized controlled trials, non‐randomized controlled trials, and quasi‐experimental studies were eligible study designs. All settings (e.g., leisure centers, health clubs, and primary care) and delivery formats (e.g., group, individual, face‐to‐face, and digital) were included. Studies focusing on the primary prevention of weight gain/obesity were not included. The presence of the following obesity comorbidities was not an exclusion criterion: type 2 diabetes, hypertension, dyslipidemia, metabolic syndrome, liver disease (NAFLD/NASH), and osteoarthritis (rf summary paper for details). No minimum intervention length criterion was applied. Comparators included no intervention, standard care, or dietary interventions without a PA practice/counseling component.

Abstracts and full texts were assessed for eligibility independently by two authors (EVC and JE) with uncertainty regarding eligibility discussed among authors.

### 2.3 Data extraction and synthesis

Data was extracted by two authors (EVC and JE) using standardized forms. The following characteristics were retrieved from each article: reference, study design, number of participants included in intervention and control groups, population characteristics (age, BMI, % female, comorbidities for intervention and control groups), trial characteristics (program description and comparison, theoretical rationale and BCTs, delivery format, length, and follow‐up), outcomes, and main results.

BCTs were coded as present or absent using the BCTTv1 taxonomy^6^ and the new MBCT taxonomy^9^ for all intervention and control conditions. A BCT was only coded when there was clear evidence of its direct application to PA. The total number of (M)BCTs used in each active condition was also registered, as was the congruence between the theoretical basis and the used (M)BCTs in each intervention. Two reviewers (EVC and JE) independently coded all included papers, and disagreements were solved by consensus. Full protocols and related papers were consulted when available.

### 2.4 Quality assessment

To assess study quality, we used the tool developed by the National Heart, Lung, and Blood Institute (NHLBI, USA) that has been previously used for defining guidelines for the management of obesity.^23^ The original assessment form for controlled/comparative trials was used. This form comprises 14 items answered on a yes/no basis. Four of these items represented fatal flaws if answered “No/Not reported/Can't determine”: (i) randomization, (ii) dropout rate <20%, (iii) valid/reliable outcome measures, and (iv) intent‐to‐treat analysis. A global rating was determined based on the number of fatal flaws: good quality (0 *fatal flaws*), fair quality (1 *fatal flaw*), or poor quality (≥2 *fatal flaws*). Quality assessment was conducted independently by two reviewers, and disagreement was resolved through discussion (with a third author where necessary).

### 2.5 Data analysis

Meta‐analyses using pre–post changes in PA in all active (using ≥1 BCT) intervention and control conditions were conducted using the Comprehensive Meta‐Analysis (CMA) Software Version 3.3.070.^24^ This type of meta‐analysis is considered more reliable to assess differences in subgroups for intervention characteristics like BCTs^25^ and allowed the inclusion of more trials and testing the moderating role of more BCTs. Random effects models were chosen due to the considerable heterogeneity among studies in interventions' length, content (i.e., BCTs and theoretical background), and sample size. Separate meta‐analyses were conducted for digital and face‐to‐face interventions. Subgroup analyses were used to assess the impact of study quality and type of PA measures (i.e., objective vs. self‐reported).

PA outcomes were assessed with different instruments (some objectively, others by self‐report) and therefore combined as standardized mean differences (SMDs). Effect sizes were computed based on pre‐ and post‐intervention scores for each active arm using means (M), standard deviations (SD), and sample sizes for each group or based on the mean difference (and SD of the difference). When these data were missing, effect sizes were obtained from alternative parameters (e.g., means and standard errors or interquartile ranges). Effect sizes were interpreted according to Cohen's^26^ guidelines (values of 0.20, 0.50, and 0.80 for small, medium, and large effect sizes, respectively). The 95% CI, *Z*‐values, and corresponding *p*‐values were considered as indicators of the significance of the effect. We also inspected the standard residuals for outliers (>1.96).

Heterogeneity was tested using the *I* ^*2*^ statistic^27^ and the Cochran's *Q* statistic.^28^ The *I* ^*2*^ ranges from 0 to 100%, where values of 25%, 50%, and 75% reflect low, moderate, and high heterogeneity, respectively.^27^ The Cochran's *Q* statistic demonstrates that studies do not share a common effect size (i.e., there is heterogeneity) when a significant *p*‐value (<0.05) is found.^29^

The potential for publication bias was subjectively assessed by inspecting funnel plots for asymmetry. They were quantitatively assessed using Egger's test^28^ and Duval and Tweedie's trim‐and‐fill method^30^ when 10 or more studies were available per meta‐analysis and no substantial heterogeneity was present, because the power is too low to distinguish chance from real asymmetry.^31^

To explore heterogeneity within main effects analyses, moderator effects of BCTs were investigated using restricted maximum likelihood random effects meta‐regressions. Univariate meta‐regressions were conducted to examine the association between individual BCTs that were present (vs. not) and the effect of the number of BCTs used. Predefined analyses of long‐term outcomes and theory‐congruent BCT sets were not possible due to the small number of studies analyzing long‐term (i.e., five of 12 with available data for meta‐analyses) and the large number of different BCT combinations used under one same theoretical framework (including some not belonging to that framework). The association between covariates and PA effect size was investigated using regression coefficients (*β*) and adjusted *R* ^2^ (used as a measure of the proportion of variance accounted for by the covariate); *β* values > 0.10 in conjunction with an adjusted *R* ^2^ of >10% indicated an important association.^7^ Multivariate meta‐regressions including significant BCTs were also conducted. Meta‐regressions were only performed when there was evidence of substantial heterogeneity (*I* ^*2*^ ≥ 50%) and ≥ 10 trials per analysis^25^ and at least two trials using a BCT to minimize the impact of single trials.

Sensitivity analyses were carried out to explore the impact of risk of bias on effect sizes by repeating primary analyses with the exclusion of studies/arms with (i) poor quality, (ii) detected as outliers (>1.96), or (iii) using self‐reported PA measures.

## 3 RESULTS

**FIGURE 1:**
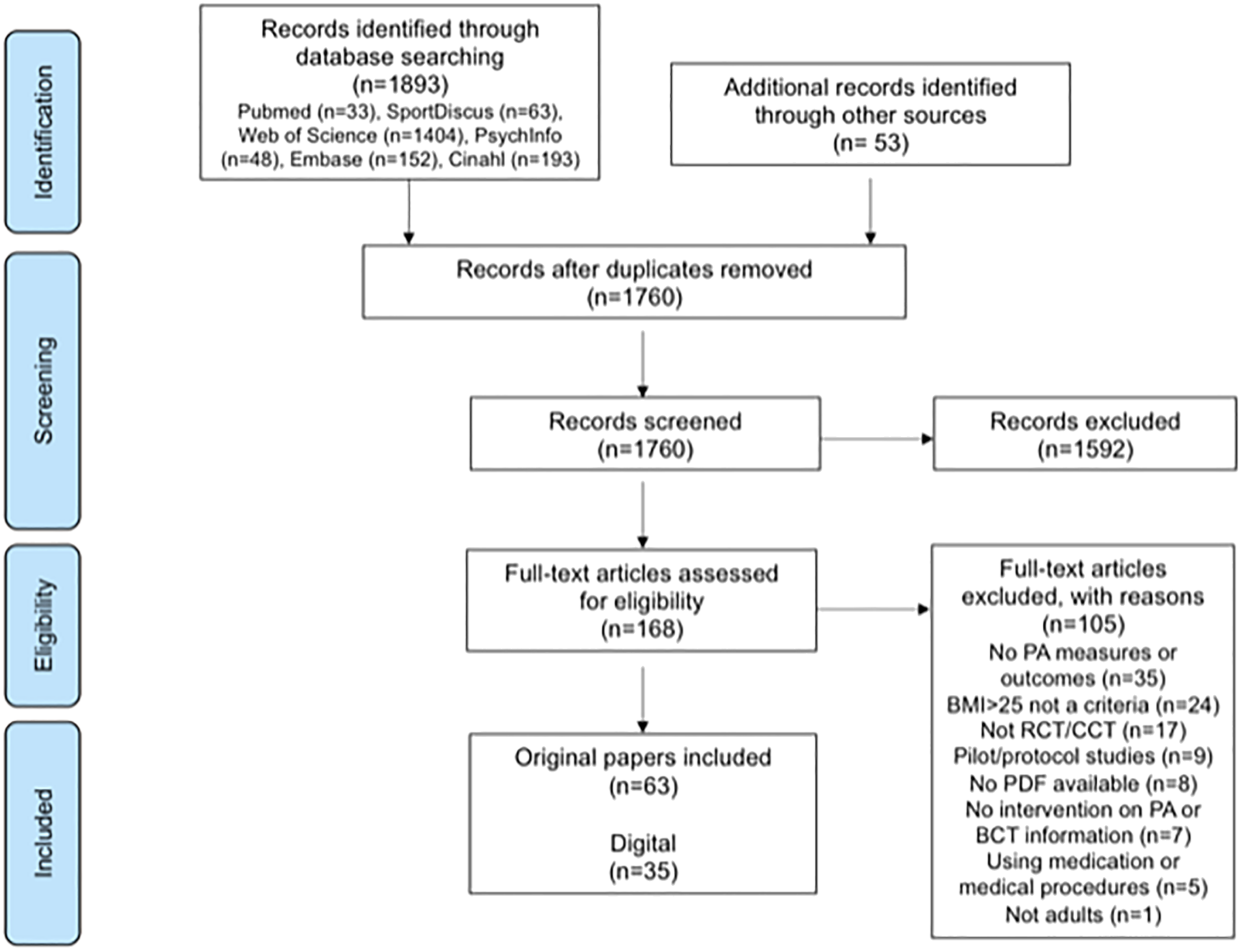
Systematic review flow diagram

Figure 1 illustrates the systematic review flow diagram. The database search yielded 1760 articles after duplicates were removed, 1592 of which were eliminated based on titles and abstracts alone. The full text was retrieved from 168 articles, and 63 satisfied the inclusion criteria. Sixty‐two original studies, corresponding to 104 distinct intervention arms using at least one BCT (*k* = 104), were included in this systematic review. From these, 35 studies (*k* = 59) referred to digital trials, and 28 studies (*k* = 45) to face‐to‐face trials.

### 3.1 Study characteristics

The characteristics of the included studies are presented in Table S2. Studies were published between 1999 and 2019. Studies included randomized (*n* = 59) or non‐randomized (*n* = 3) controlled trials. These 62 studies represent over 12,854 participants (6240 from digital trials and 6614 from face‐to‐face trials). The number of participants in each study ranged from 36 to over 1300 (median, 114 participants). Mean age of trial participants ranged from 20 to 58 years in digital trials (median, 49 years) and from 20 to 63 years in face‐to‐face trials (median, 48 years). Most participants were female (77% average). Eight studies recruited women only, and two recruited men only. Mean BMI ranged from 28 to 43 kg/m^2^ in digital interventions (median, 33 kg/m^2^) and from 29 to 39 kg/m^2^ in face‐to‐face trials (median, 33 kg/m^2^). Twelve studies (19%) included participants with comorbidities: metabolic syndrome,^32^, ^33^ type 2 diabetes,^34^, ^35^, ^36^, ^37^, ^38^, ^39^, ^40^ and hypertension.^35^, ^38^, ^41^, ^42^, ^43^

PA was targeted in 11 trials (17%), six digital trials (*k* = 13), and five face‐to‐face trials (*k* = 11); PA and diet were targeted in 49 trials (78%), 26 digital studies (*k* = 42), and 23 face‐to‐face studies (*k* = 33), PA and sedentary behavior in two studies (3%; *k* = 3), and PA and weight in one study (2%; *k* = 2). Regarding control groups, 30 studies involved a standard care control (48%), and 18 a waiting list control (29%). Fourteen studies were comparative, involving two or more active conditions (23%).

Length ranged from 2 to 78 weeks (median, 26 weeks) overall. Only 23 studies (37%; five digital) included a follow‐up period of variable duration (median, 39 weeks), which was generally longer in face‐to‐face trials (median, 78 weeks). Fifteen studies (24%) used an objective measure of PA (nine digital), 41 studies (65%) used a self‐reported PA measure (19 digital), and seven (11%) used a mixture of objective and self‐reported measures (three digital).

Regarding changes in PA in active conditions (using ≥1 BCT), 54% and 69% of active conditions reported significant changes in self‐reported and objective PA, respectively. In digital interventions, 56% and 74% active conditions reported significant changes in self‐reported and objective PA, respectively. In face‐to‐face interventions, 52% and 63% of active conditions reported significant changes in self‐reported and objective PA, respectively.

Forty‐five studies (71%) reported to be grounded on one or more theoretical frameworks. Only 15 of the 45 trials reported using a single theory. Theoretical basis was highly variable with social cognitive theory and self‐regulation theory, alone (*n* = 25; *n*_digital_ = 17 and 14, respectively) or in combination (*n* = 12; *n*_digital_ = 8) used most often.

### 3.2 Coding of BCTs

Interventions were typically complex with the number of identified techniques ranging from 1 to 21 in digital interventions (median, eight BCTs) and from 1 to 26 in face‐to‐face interventions (median, six BCTs). The most frequently used BCTs in digital interventions were *goal setting (behavior)*, *self‐monitoring of behavior*, *feedback on behavior*, *problem solving*, *social support*, *instruction on how to perform behavior*, *prompts/cues*, *self‐monitoring of outcomes*, *graded tasks*, and *information about health consequences*. When considering face‐to‐face interventions, two additional BCTs were frequently used—*behavioral practice/rehearsal* and *social reward*, whereas *prompts/cues* and *information on health consequences* were less frequently used. MBCTs were used in few studies (*n* = 9), and only four studies used nine or more of these techniques (of 21 possible MBCTs).^44^, ^45^, ^46^, ^47^ The most frequently used were *provide choice*, *prompt identification and seek available social support*, *help develop a clear concrete action plan*, *elicit perspectives on condition/behavior*, *and address obstacles for change*. Overall, 39 techniques were used less than five times (Table 1). Forty techniques were not identified in any intervention descriptions.

**TABLE 1:** Number of times each BCT was used in interventions aimed at increasing PA (overall, in digital and in face‐to‐face format)

	Interventions		
Behavior change techniques	Overall	Digital	Face‐to‐face
Goal setting (behavior)	84	46	38
Self‐monitoring of behavior	77	44	33
Feedback on behavior	50	34	17
Problem solving	50	27	23
Social support (unspecified)	40	27	13
Instruction on how to perform the behavior	38	20	18
Prompts/cues	36	33	3
Self‐monitoring of outcomes of behavior	30	18	12
Graded tasks	29	15	14
Information about health consequences	27	16	11
Behavioral practice/rehearsal	27	7	20
Social reward	26	14	12
Demonstration of behavior	25	10	15
Action planning	20	8	10
Goal setting (outcome)	20	8	12
Feedback on outcomes of behavior	18	12	6
Credible source	16	13	3
Discrepancy between current behavior and goal	13	3	10
Review behavior goals	13	9	4
Social comparison	12	7	5
Nonspecific reward	8	4	4
*Prompt identification and seek available social support*	8	4	4
*Provide choice*	8	6	2
Social incentive	8	5	3
Pros and cons	7	2	5
Self‐reward	7	3	4
Material reward (behavior)	6	4	2
*Help develop clear concrete action plan*	6	2	4
Framing/reframing	6	2	4
*Elicit perspectives on condition/behavior*	6	1	5
*Address obstacles for change*	6	1	5
*Assist in setting optimal challenge*	5	3	2
*Offer relevant, clear, constructive feedback*	5	2	3
*Use empathic listening*	5	2	3
Material incentive^a^	5	5	0
Avoid/reduce exposure to cues for the behavior^a^	4	4	0
Reduce negative emotions^b^	4	0	4
Behavioral contract	4	3	1
Reward approximation	4	3	1
Self‐incentive	4	3	1
Focus on past success	3	1	2
Reward (outcome)	3	1	2
Non‐specific incentive	3	2	1
Social support (emotional)	3	1	2
*Use non‐controlling informational language*	3	0	3
*Explore life aspirations and values*	3	1	2
*Acknowledge and respect perspectives and feelings*	3	1	2
*Providing opportunities for ongoing support*	3	2	1
*Clarify expectations*	3	1	2
*Promote self‐monitoring*	3	1	2
Body changes^a^	2	2	0
Behavior cost^a^	2	2	0
Monitoring emotional consequences^b^	2	0	2
Information about others approval^b^	2	0	2
Commitment	2	1	1
*Encourage person to experiment and initiate behavior*§	2	0	2
*Show unconditional regard* ^b^	2	0	2
Incentive (outcome)^a^	1	1	0
Comparative imagining of future outcomes^a^			
Valued self‐identity^a^	1	1	0
Verbal persuasion about capability^a^	1	1	0
Vicarious consequences^a^	1	1	0
Monitoring of behavior by others without feedback^b^	1	0	1
Monitoring of outcomes by others without feedback^b^			
Information about antecedents^b^	1	0	1
Information about emotional consequences^b^	1	0	1
Distraction^b^	1	0	1
Restructuring the social environment^b^	1	0	1
Adding objects to the environment^a^	1	1	0
Identification of self as role model^b^	1	0	1
Identity associated with changed behavior^b^	1	0	1
Self‐talk^b^	1	0	1
*Provide a meaningful rationale* ^b^	1	0	1
*Explore ways of dealing with pressure* ^b^	1	0	1

*Notes*: BCTs are organized by frequency of use (in descent order) in the overall interventions. MBCTs are identified in italic. BCTs that were not identified in any intervention are not included in this table.

^a^ BCTs used only in digital interventions.

^b^ BCTs used only in face‐to‐face interventions.

Regarding the use of set combinations of techniques, no two trials used exactly the same BCT combination; not even any of the 50 trials reporting to be theory based. Inspection of theory‐congruent combinations of techniques based on the classification provided by Abraham and Michie's^48^ evidenced a lack of coherence among trials reporting to have used the same theoretical framework. Also, few (if any) interventions used all the BCTs that were congruent with self‐regulation theory, social cognitive theory, operant conditioning, or self‐determination theory. Moreover, the chosen BCT combinations generally tapped into several theories, beyond the one that was mentioned. Therefore, it was not possible to isolate a certain set of theory‐congruent BCTs and test its effectiveness in meta‐analyses. Finally, some studies (*n* = 12) were not explicit regarding the underlying theoretical background or did not use any.

Regarding the level of detail provided in the papers with respect to the techniques used in the interventions, 45 papers (*k* = 77) presented a detailed description of the interventions and BCTs, explaining how the techniques were implemented. The remaining 17 papers (*k* = 27) did not include a proper description of how the reported BCTs were used. Only six papers (10%) included the BCTs as listed in the taxonomies.^36^, ^46^, ^49^, ^50^, ^51^, ^52^

### 3.3 Study quality

Of the 62 studies identified as relevant for this review, the methodological quality of 15 studies was rated as good, 26 were classified as fair, and 21 were rated as poor. Regarding the main flaws, three studies did not use randomized controlled designs, 26 presented dropout rates above 20%, 26 studies did not use objective measures of PA, and 20 did not perform intent‐to‐treat analysis (Table S3).

### 3.4 Intervention effects on PA

A total of 3115 participants from digital trials and 2556 from face‐to‐face trials were included in the meta‐analysis. The SMD in PA was 0.42 (95% CI 0.28–0.57, *k* = 39) in digital interventions and 0.78 (95% CI 0.51–1.01, *k* = 35) in face‐to‐face interventions, representing medium–high significant effect sizes. Heterogeneity was high in digital (*I* ^2^ = 92%; *Q* = 498, *p* < 0.001) and face‐to‐face interventions (*I* ^2^ = 96%; *Q* = 912, *p* < 0.001). Using objective (vs. self‐reported) PA measures resulted in a higher pooled effect size in digital interventions (0.73, 95% CI 0.38–1.09, *k* = 16 vs. 0.25, 95% CI 0.15–0.35, *k* = 23), whereas in face‐to‐face interventions, it resulted in a lower and nonsignificant effect size (0.04, 95% CI −0.22–0.29, *k* = 12 vs. 1.20, 95% CI 0.92–1.49, *k* = 24). There was evidence of publication bias based on the visual inspection of the funnel plot, but due to the large heterogeneity observed in both digital and face‐to‐face interventions, there was not enough power to differentiate chance from real asymmetry.^31^

**FIGURE 2:**
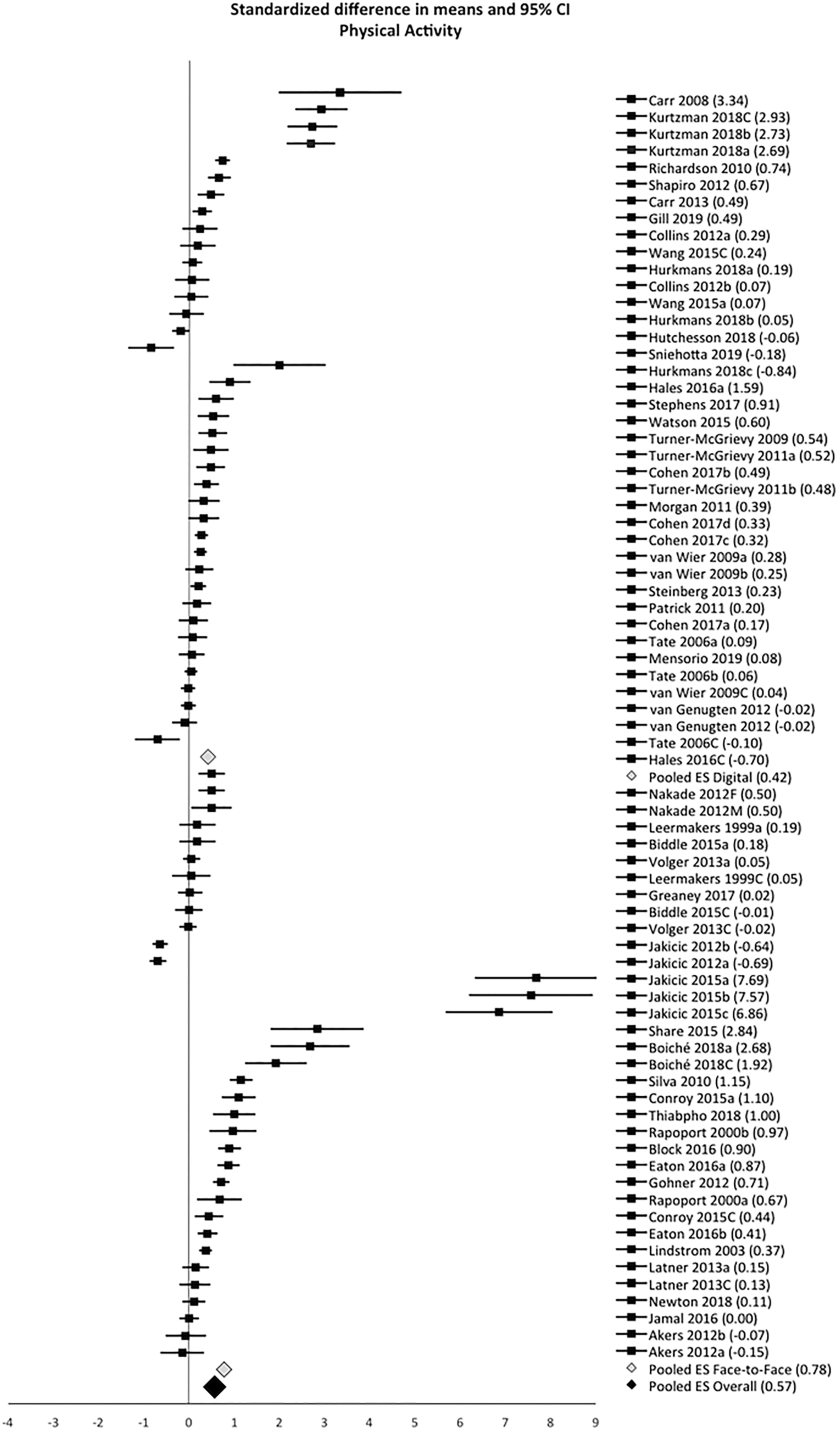
Forest plot representing all active arms included in the meta‐analysis. Digital arms are depicted on top and face‐to‐face arms on the bottom, organized by type of PA measure (starting with objective PA and then self‐reported PA) and in descending order, from the highest to the lowest effect size. Three pooled effect sizes are depicted: in gray, for digital interventions and for face‐to‐face interventions; in black, for the overall interventions

Study quality led to significant differences in PA pooled effect sizes. Sensitivity analysis removing poor quality studies resulted in a ~10‐point increase in effect sizes (digital interventions: 0.55, 95% CI 0.47–0.86, *k* = 27; face‐to‐face interventions: 0.88, 95% CI 0.55–1.22, *k* = 23). On the other hand, excluding identified outliers decreased the effect sizes (digital interventions: 0.27, 95% CI 0.18–0.37, *k* = 33; face‐to‐face interventions: 0.39, 95% CI 0.25–0.52, *k* = 26). Figure 2 depicts the forest plot showing PA effect sizes with 95% CI for digital, face‐to‐face, and overall interventions. For control purposes, a meta‐analysis including pre–post changes in non‐active controls was also conducted, resulting in a negligible, nonsignificant pooled effect size (0.06, 95% CI −0.07–0.18, *k* = 24), slightly higher in face‐to‐face interventions (0.12, 95% CI 0.02–0.23, *k* = 11).

### 3.5 Moderator analyses of BCTs

Twenty‐five moderator analyses were conducted to investigate differences in PA pooled effect size according to the presence or absence of BCTs in digital or face‐to‐face interventions. The effect of the number of BCTs used was also explored as a moderator. Meta‐regression results are shown in Table 2.

**TABLE 2:** Results from meta‐regression analysis exploring moderating effects of behavior change techniques on PA outcomes

	Univariate meta‐regressions					Multivariate meta‐regressions			Sensitivity analysis		
Study characteristics^a^	*β*	95% CI	*p*‐Value	Adj. *R* ^2^%	*β*	95% CI	*p*‐Value	Adj. *R* ^2^ %	No poor‐quality studies	No outliers	Only objective PA measures
*Digital interventions (k = 39)*											
Goal setting behavior	0.89	0.36, 1.42	0.001	20	1.10	0.06, 2.15	0.033	49	✓		
Goal setting outcome	0.76	0.18, 1.35	0.011	15					✓		
Graded tasks	0.87	0.23, 1.51	0.008	17					✓	✓	
Social incentive	2.37	1.50, 3.25	<0.001	51	1.72	0.71, 3.04	0.010		✓		✓
Self‐monitoring of behavior	−1.04	−1.67, −0.42	0.001	23					✓^b^		✓^b^
*Face‐to‐face interventions (k = 35)*											
Behavioural practice and rehearsal	1.83	0.72, 2.95	0.001	22					✓	✓^c^	✓^d^

*Notes*: Univariate meta‐regression: Twenty‐five behavior change techniques were tested. Multivariate meta‐regressions: Significant predictors were added simultaneously and tested as predictors.

Abbreviations: adj. *R* ^2^ %, adjusted proportion of between study variance explained by behavior change techniques; CI, confidence interval; *β*, estimated meta‐regression coefficient.

^a^ Only predictors with significant effects (*p* < 0.05) and adjusted R^2^ above 0.10 are reported.

^b^ Self‐monitoring of behavior remained a significant negative moderator in multivariate analysis.

^c^ Behavioral practice and rehearsal approached significance (*p* = 0.057). Discrepancy between goals and behavior and pros and cons emerged as significant positive moderators.

^d^ Seven techniques were significant in univariate analysis, but only behavioral practice and rehearsal and non‐specific reward (negative effect) were significant in multivariate analysis.

#### 3.5.1 Effective PA‐specific BCTs in digital interventions (*k* = 39)

Four BCTs showed significant positive moderator effects on PA: *goal setting behavior* (*β* = 0.89, *p* = 0.001, adjusted *R* ^2^ = 0.20), *goal setting outcome* (*β* = 0.76, *p* = 0.011, adjusted *R* ^2^ = 0.15), *graded tasks* (*β* = 0.87, *p* = 0.008, adjusted *R* ^2^ = 0.17), and *social incentive* (*β* = 2.37, *p* < 0.001, adjusted *R* ^2^ = 0.51). One BCT showed significant negative moderator effects on PA, *self‐monitoring of behavior* (*β* = −1.04, *p* = 0.001, adjusted R^2^ = 0.23). In multivariate meta‐analysis (adjusted *R* ^2^ = 0.49), only *goal setting behavior* (*β* = 1.10, *p* = 0.039) and *social incentive* (*β* = 1.72, *p* = 0.010) remained significant. Sensitivity analyses removing poor quality studies led to similar findings in univariate meta‐regression, but in multivariate analysis, only *self‐monitoring of behavior* remained significant (*β* = −1.17, *p* = 0.021, adjusted *R* ^2^ = 0.56). When removing outliers, a significant positive moderator effect of *graded tasks* was observed (*β* = 0.32, *p* = 0.005, adjusted *R* ^2^ = 0.28), and when including objective PA measures only, *social incentive* positively (*β* = 2.21, *p* = 0.003, adjusted *R* ^2^ = 0.41) and *self‐monitoring of behavior* negatively (*β* = −2.53, *p* < 0.001, adjusted *R* ^2^ = 0.86) emerged as significant moderators, but only the second remained significant in the multivariate meta‐regression.

#### 3.5.2 Effective PA‐specific BCTs in face‐to‐face interventions (*k* = 35)

One BCT showed significant positive moderator effects on PA, *behavioral practice and rehearsal* (*β* = 1.83, *p* = 0.001, adjusted *R* ^2^ = 0.22). Sensitivity analyses excluding poor quality studies led to identical findings. When removing outliers*,* two other BCTs (used in very few active arms) were significantly associated with higher PA pooled effect sizes, *discrepancy between goals and behavior* (*β* = 0.48, *p* = 0.016, adjusted *R* ^2^ = 0.22) and *pros and cons* (*β* = 0.44, *p* = 0.034, adjusted *R* ^2^ = 0.16), and *behavioral practice and rehearsal* approached significance (*β* = 0.30, *p* = 0.057, adjusted *R* ^2^ = 0.08). In multivariate meta‐analysis, none was significant. When including objective PA measures only, four BCTs positively moderated effects on PA: *behavioral practice and rehearsal* (*β* = 0.47, *p* = 0.028, adjusted *R* ^2^ = 0.31), *action planning* (*β* = 0.49, *p* = 0.039, adjusted *R* ^2^ = 0.27), *social support* (*β* = 0.62, *p* = 0.005, adjusted *R* ^2^ = 0.42), and *social reward* (*β* = 0.62, *p* = 0.005, adjusted *R* ^2^ = 0.42). Three BCTs negatively moderated effects on PA: *problem solving* (*β* = −0.42, *p* = 0.044, adjusted *R* ^2^ = 0.25), *goal setting outcome* (*β* = −0.74, *p* = 0.036, adjusted *R* ^2^ = 0.26), and *nonspecific reward* (*β* = −0.63, *p* < 0.001, adjusted *R* ^2^ = 0.57). Of these, only two remained significant in multivariate meta‐analysis (adjusted *R* ^2^ = 0.93): *behavioral practice and rehearsal* (*β* = 0.33, *p* = 0.020) and *nonspecific reward* (*β* = −0.54, *p* < 0.001).

## 4 DISCUSSION AND CONCLUSION

The current systematic review and meta‐analysis applied reliable BCT taxonomies to identify effective BCTs within complex behavioral interventions aimed at increasing PA in adults with overweight or obesity, distinguishing digital from face‐to‐face delivered interventions. This review was also one of the first to employ the recent motivational BCT taxonomy^9^ to account for the specific effect of interpersonal style techniques, which were previously argued to be missing from available taxonomies.^53^ In line with previous research,^8^, ^12^ meta‐analysis results showed that behavior change interventions for increasing PA in adults with overweight or obesity were moderately effective both in digital and face‐to‐face interventions, considering the negligible, nonsignificant pooled effects on PA observed in non‐active control groups (using no BCTs). The observed heterogeneity was generally high and also coherent with previous research.

A large heterogeneity in intervention content (i.e., sets of BCTs used) was also observed, with none of the interventions reporting the same combination of techniques. An inspection of theory‐congruent combinations of techniques also evidenced a lack of coherence (i) between the theoretical framework and the intervention content (i.e., some used BCTs were not in the scope of the theory[ies] reported to be used) and (ii) among trials based on the same theory (i.e., studies with the same theoretical underpinnings did not use the exact same combination of techniques). These aspects are likely to explain part of the variability in PA outcomes.

The total number of techniques used did not show significant moderating effects in either digital or face‐to‐face interventions, suggesting that the quality rather than the quantity of techniques might be more important to produce changes in PA outcomes. Similarly, Dombrowski et al.^12^ were not able to find a dose–response relationship between the number of techniques and PA outcomes in adults with obesity and additional comorbidities. Michie et al.^7^ have also found similar results in healthy adults, arguing that combinations of large numbers of techniques might be illusive, weakening the impact of the most effective techniques and compromising the fidelity of delivery. In contrast, Samdal et al.^8^ did find a positive association with intervention effectiveness. These contradictory findings highlight the need for further investigation on this matter.

Moderator analysis revealed that the most effective BCTs are likely to differ between digital and face‐to‐face interventions. Regarding the first delivery format, meta‐regressions showed that four BCTs positively moderated PA changes—*goal setting (behavior)*, *goal setting (outcome)*, *graded tasks*, *and social incentive*—whereas one BCT negatively moderated PA changes, *self‐monitoring of behavior*. From these techniques, only *goal setting (behavior)* and *social incentive* remained significant in multivariate meta‐regressions. Sensitivity analysis generally confirmed these findings, with one or more of these techniques emerging as significant. No previous review has systematically and quantitatively examined effective BCTs specifically in digital interventions in adults with overweight and obesity. A prior systematic review reporting on Internet use to promote health behavior change in the general population has also identified goal setting as an effective technique,^17^ as did Michie et al.^7^ in PA and healthy eating interventions. A recent meta‐review^54^ of self‐regulatory BCTs to promote weight loss and related behaviors (i.e., PA and healthy eating) suggested mixed results. From the three examined reviews, two found an association between improved outcomes and the inclusion of goal setting.

The most striking and unexpected finding in the current review was the negative moderating effect of *self‐monitoring of behavior*, as this technique is generally recognized as a valuable and positive asset to foster health behavior change.^7^, ^8^, ^13^, ^55^ However, in a recent systematic meta‐review, Hennessy et al.^56^ found that the quality of the evidence synthesis for this intervention component was quite variable across health behaviors and dependent on the particular population and health outcome of study. For instance, French et al.^57^ found that *self‐monitoring of behavior* was associated with lower levels of PA in older adults, and Webb et al.^17^ found a small and nonsignificant effect in digital interventions for the general population. Also, prior research revealed that *self‐monitoring* needs to be used consistently (e.g., once a week) to generate better exercise and weight outcomes,^58^ something that can be achieved by including additional features like individualized feedback or reminders in web‐based interventions.^59^ The type of tools used to self‐monitor PA has also been shown to affect the effectiveness of this BCT, with newer self‐monitoring technologies apparently more effective.^60^, ^61^

In face‐to‐face interventions, *behavioral practice and rehearsal* was consistently identified as a positive moderator of PA outcomes in this population, including in sensitivity analysis. This finding is in line with prior reviews suggesting that the use of this technique, specifically to target PA, significantly moderated intervention effects on weight^12^, ^13^ and seems to be associated with positive changes in behavioral intentions and stages of change for PA in face‐to‐face interventions, which were in turn related with increases in PA.^55^ Another recent review on PA interventions for inactive healthy adults also found that intervention effectiveness was associated with the use of *behavior practice and rehearsal*.^62^ A few additional BCTs came up as significant in sensitivity analysis but were used in very few interventions and/or appeared only once (see legend in Table 2).

Motivational techniques were seldom used, precluding the testing of their effectiveness in meta‐regression analyses. Nevertheless, some MBCTs were frequently present in interventions that successfully changed PA, especially in face‐to‐face interventions: *prompt identification and seek available social support*, *elicit perspectives on condition/behavior*, *help develop clear concrete action plan*, *and provide choice* (data not shown). Also, Samdal et al.^8^ have found that using a person‐centered approach and an autonomy‐supportive counseling were associated with sustained behavior adherence. The interpersonal style of delivery (reflected in MBCTs) has been argued to be as important as the intervention techniques used^53^ and capable of increasing the efficacy of contingency‐based BCTs like rewards, incentives, or threats to foster behavioral adherence, if these techniques are delivered in an autonomy‐supportive manner.^63^ Further research and examination of MBCTs is clearly needed to determine their effectiveness.

In the current systematic review, only a few techniques were associated with more successful PA outcomes among people with overweight or obesity. However, important considerations must be made when interpreting these findings. First, nonsignificant moderating effects of specific BCTs do not mean that those techniques do not have an effect, but rather that their effect might have been suppressed or masked by other BCTs used in combination. Likewise, significant moderator effects identified herein cannot guarantee that using those particular BCTs will generate greater improvements in PA. This could simply be due to chance alone, given the multiple tests conducted, but also to contextual factors or other intervention characteristics that might also have affected the outcomes.^64^ Moreover, BCTs cannot be isolated from each other and were implemented in very diverse combinations, possibly resulting in different interaction effects.

Additionally, intervention descriptions were not always sufficiently detailed and precise regarding the employed BCTs, which might have resulted in the misidentification or incorrect coding of BCTs in approximately 30% of the studies included in the current review, which generally lacked information on how the techniques were implemented and/or did not use the BCT names listed in the taxonomies. This issue has been observed in prior reviews^12^, ^65^ and in a recent scoping review.^64^ Finally, there is an issue of implementation fidelity, as a technique or a combination of techniques might not have been used as planned a priori, or it might have been improperly delivered, which would naturally influence its effectiveness.

In conclusion, effective BCTs for increasing PA in adults with overweight and obesity in digital and face‐to‐face interventions seem to differ. Evidence suggests that using *goal setting*, *social incentive*, and *graded tasks* might be considered to improve PA outcomes in digital interventions. It might also be useful to avoid inconsistent *self‐monitoring of behavior* and opt for newer, more objective self‐monitoring technologies to increase compliance with the use of behavioral self‐monitoring. Regarding face‐to‐face interventions, evidence suggests that it might be useful to prompt *behavioral practice and rehearsal* to obtain more favorable PA outcomes. Still, these conclusions should be interpreted in light of the limitations of the available evidence. Michie et al.^64^ recently concluded that only weak conclusions could be drawn regarding the effectiveness of specific BCTs or BCT combinations, provided that all available methods for identifying effective BCTs, linked to a given health behavior and context, have important limitations. Further research, including more detailed and standardized descriptions of intervention content (i.e., BCTs) and measures of implementation fidelity, is clearly required and likely to have an impact on the moderating effects found in the current review. There is also insufficient evidence to examine the effectiveness of most BCTs included in the two coding taxonomies; even more so if we consider long‐term effectiveness, as less than half of the studies included a follow‐up and of highly variable length. No consistent (theory‐congruent) combinations of BCTs for increasing PA in this population were identified, calling for greater standardization at this level as well. In spite of the limitations in the available evidence and the low to moderate certainty in the observed results, the EASO PA Working Group thought it was important to provide clinical guidance and make the abovementioned recommendations, as more benefits than harms are to be expected. Further research might have an impact on these results and is therefore recommended.

## CONFLICT OF INTEREST

No conflict of interest statement.

## AUTHOR CONTRIBUTIONS

EC and JE performed the literature search, study selection, data extraction, and quality assessment. All authors participated in the interpretation of data. EC and JE drafted the manuscript, and authors critically revised the manuscript.

## Supporting information

**Table S1.** Pubmed Search Strategy
Table S2. Characteristics of included studies.
Table S3. Quality of included original controlled studies.
