# Supplementary material for: Effective behavior change techniques to promote physical activity in adults with overweight or obesity: A systematic review and meta‐analysis
Source: Obes Rev. 2021 May 5;22(Suppl 4):e13258. doi: 10.1111/obr.13258 (PMC8365685; doi:10.1111/obr.13258)
Supplement: Supplementary file 1 — Table S1. Pubmed Search Strategy Table S2. Characteristics of included studies. Table S3. Quality of included original controlled studies. [file OBR-22-e13258-s001.pdf]

Effective Behaviour Change Techniques to Promote Physical Activity in Adults with Overweight or Obesity: A Systematic Review and Meta-Analysis

Authors: Eliana V. Carraça[1], Jorge Encantado[2], Francesca Battista[3], Kristine Beaulieu[4], John E. Blundell[4], Luca Busetto[5,6], Marleen van Baak[7], Dror Dicker[5,8], Andrea Ermolao[3], Nathalie Farpour-Lambert[5,9], Adryan Pramono[7], Euan Woodward[5], Alice Bellicha[10,11], Jean-Michel Oppert[11]*

[1] CIDEFES, Universidade Lusófona de Humanidades e Tecnologias, Faculdade de Educação Física e Desporto. Lisboa, Portugal.

[2] APPsyCI – Applied Psychology Research Center Capabilities & Inclusion; ISPA - University Institute.

[3] Sport and Exercise Medicine Division, Department of Medicine, University of Padova, Padova, Italy.

[4] Appetite Control and Energy Balance Group (ACEB), School of Psychology, Faculty of Medicine and Health, University of Leeds, Leeds, LS2 9JT, UK

[5] European Association for the Study of obesity (EASO), Obesity Management Task Force (OMTF).

[6] Department of Medicine, University of Padova, Padova, Italy.

[7] NUTRIM School of Nutrition and Translational Research in Metabolism, Department of Human Biology, Maastricht University Medical Centre+, Maastricht, The Netherlands

[8] Department of Internal Medicine D, Hasharon Hospital, Rabin Medical Center, Sackler School of Medicine, Tel Aviv University, Tel Aviv, Israel

[9] Obesity Prevention and Care Program Contrepoids; Service of Endocrinology, Diabetology, Nutrition and Patient Education, Department of Internal Medicine, University Hospitals of Geneva and University of Geneva, Switzerland.

[10] Sorbonne University, INSERM, Nutrition and obesities; systemic approaches (NutriOmics), Paris, France.

[11] University Paris-Est Créteil, UFR SESS-STAPS, Créteil, France

[12] Assistance Publique-Hôpitaux de Paris (AP-HP), Pitié-Salpêtrière hospital, Department of Nutrition, Institute of Cardiometabolism and Nutrition; Sorbonne University, France.

Table S1. Pubmed Search Strategy

1	(obesity OR overweight OR obese) AND adults[Title/Abstract] (31287)
2	(lifestyle intervention OR behavioral intervention OR behavioural intervention OR behavior change intervention OR behaviour change intervention OR web-based OR internet OR digital OR ehealth OR mhealth OR e-health OR m-health OR e&mhealth OR e&m-health OR application OR mobile OR technolog* OR computer*)[Title/Abstract] (1965936)
3	(physical activit* OR exercise OR sedentary time OR sedentary lifestyle OR sitting time OR sedentary behavior OR sedentary behaviour)[Title/Abstract] (336212)
4	(clinical trial OR randomised controlled trial OR randomized controlled trial OR)[Title/Abstract] (718393)
5	1 AND 2 AND 3 AND 4 (149)
6	5 AND Humans[Mesh] AND English[lang] AND adult[MeSH]

Table S2. Characteristics of included studies.

Authors, Year	Study Design	Sample	Intervention				Outcomes, Instruments & Time points	Physical Activity Changes
			Description	Theory & Strategies	Mode of Delivery	Length + Follow-up		
Adams et al., 2017	RCT (2x2 factorial design)	IG1: 25 participants (88% women); Age, 41 ± 10.2 yrs; BMI, 33.6 ± 6.3 kg/m^2<br>IG2: 24 participants (71% women); Age, 44.5 ± 10.7 yrs; BMI, 33.1 ± 6 kg/m^2<br>IG3: 24 participants (79% women); Age, 38.4 ± 8.2 yrs; BMI, 35.1 ± 5.3 kg/m^2<br>IG4: 23 participants (70 % women); Age, 40.3 ± 7.9 yrs; BMI, 34.6 ± 7.2 kg/m^2	Four factorial groups:<br>IG1: Adaptive step goals day-percentile + Immediate rewards<br>IG2: Adaptive step goals day-percentile + Delayed non-contingent rewards<br>IG3: Static step goal day + Immediate rewards<br>IG4: Static step goal day + Delayed non-contingent rewards	Goal Setting<br>Problem Solving<br>Feedback on behaviour<br>Feedback on outcomes of behaviour<br>Instruction on how to perform the behaviour<br>Information about health consequences<br>Prompts/cues<br>Graded tasks (IG1; IG2)<br>Credible source<br>Material incentive (IG1; IG3)<br>Material reward (IG1; IG3)<br>Social reward (IG1; IG3)<br>Self-incentive (IG1; IG3)<br>Self-reward (IG1; IG3)<br>Reward approximation (IG1; IG3)	Digital	4 months	Objective PA - steps per day and MVPA minutes per day (Fitbit)<br>At baseline and 4 months	Steps/day decreased at a slower rate for participants in Adaptive Goals condition than Static Goals condition.<br>Immediate Rewards condition increased steps/day at a higher rate than delayed rewards.<br>MVPA min/day decreased during intervention at about half the rate in Adaptive Goals condition compared to Static Goals condition.
Berli et al., 2018	RCT (2 arms)	123 participants; Age, 46.3 ± 13.7 yrs; BMI, 31.1 ± 5.6 kg/m^2	IG: Information leaflet with physical activity recommendations + theory-based action control intervention delivered through daily text messages<br>CG: Only the information leaflet<br>IGv2: An experimental variation in the IG involved a dyadic delivery for half of the participants and an individual delivery for the others.	Self-regulation theory<br>Goal setting (behaviour) (IG)<br>Discrepancy between current behaviour and goal (IG)<br>Self-monitoring of behaviour (IG)<br>Social support (unspecified) (IGv2)<br>Information about health consequences (IG)	Digital	14 days +14 days of follow-up	Objective PA (triaxial GT3X ActiGraph)<br>At baseline, 14 and 28 days	Mean level of PA adherence was 12% higher in the intervention group.

Authors, Year	Study Design	Sample	Intervention: Description	Intervention: Theory & Strategies	Intervention: Mode of Delivery	Intervention: Length + Follow-up	Outcomes, Instruments & Time points	Physical Activity Changes
Block et al., 2016	RCT (2 arms)	Intervention (IG): 163 participants; Age, 55 ± 8.8 yrs; BMI, 31.1 ± 4.5 kg/m^2<br><br>Control (CG): 176 participants; Age, 54.9 ± 9.1 yrs; BMI, 31.2 ± 4.3 kg/m^2	IG: Program delivered via web and email, automated interactive voice response phone calls and a supportive smartphone application. Individually tailored weekly small-step goals on diet and PA, tracking, a team system for social support and other activities are provided. Gamification.<br><br>CG: Wait-list (usual care)	Models of behaviour change research (learning theory and habit formation; cues and triggers; social cognitive theory; theory of planned behaviour)<br><br>Goal setting (behaviour) (IG)<br>Problem solving (IG)<br>Action planning (IG)<br>Discrepancy between current behaviour and goal (IG)<br>Commitment (IG)<br>Feedback on behaviour (IG)<br>Self-monitoring of behaviour (IG)<br>Self-monitoring of outcomes of behaviour (IG)<br>Social support (unspecified) (IG)<br>Social support (practical) (IG)<br>Information about health consequences (IG)<br>Prompts/cues (IG)<br>Graded tasks (IG)<br>Material reward (behaviour) (IG)<br>Social reward (IG)<br>Social incentive (IG)<br>Self-incentive (IG)<br>Self-reward (IG)<br>Reward outcome (IG)<br>Reward approximation (IG)	Digital	6 months + 6 months follow up	Self-reported PA (Block Questionnaire)<br><br>At baseline, 3 and 6 months	There were significant PA differences between intervention and control (also seen in intention-to-treat analysis).
Carr et al., 2008	RCT (2 arms)	ALED-I (IG): 14 participants; Age, 41.4 ± 3.7; BMI, 32.3 ± 1.3 kg/m^2<br><br>Control (CG): 18 participants; Age, 49.4 ± 1.7 yrs; BMI, 30.6 ± 0.8 kg/m^2	IG: Self-paced program using interactive activities and behaviour modification strategies<br><br>CG: pedometer, bimonthly received updated exercise prescription (+10% steps)	Goal setting (behaviour) (IG)<br>Problem solving (IG)<br>Review behaviour goals (IG)<br>Self-monitoring of behaviour (IG)<br>Social support (unspecified) (IG)<br>Non-specific reward (IG)<br>Avoidance/reducing exposure to cues for the behaviour (IG)	Digital	16 weeks	Objective PA - steps/day (pedometer)<br><br>At baseline and 16 weeks	The ALED-I intervention produced a significant increase in PA, but there were no significant between-group differences.

Authors, Year	Study Design	Sample	Intervention				Outcomes, Instruments & Time points	Physical Activity Changes
			Description	Theory & Strategies	Mode of Delivery	Length + Follow-up		
Carr et al., 2013	RCT (2 arms)	Intervention (IG): 23 participants (86.9% women); Age, 42.6 ± 8.9 yrs; BMI, 31.7 ± 4.9 kg/m^2<br><br>Control (CG): 17 participants (94.1% women); Age, 47.6 ± 9.9 yrs; BMI, 33.2 ± 4.5 kg/m^2	IG: Multi-component intervention to reduce daily sedentary time with access to a portable pedal machine at their worksite; a motivational website to receive tips and reminders focused on increasing active sitting through pedalling and taking breaks from sitting; and a pedometer; a software package for objective monitoring of pedal activity; real-time feedback<br><br>CG: Wait-list	Social Cognitive Theory<br><br>Goal setting (behaviour) (IG)<br>Feedback on behaviour (IG)<br>Self-monitoring of behaviour (IG)<br>Social support (unspecified) (IG)<br>Prompt/cues (IG)<br>Social reward (IG)<br>Social incentive (IG)<br>Adding objects to the environment (IG)	Digital	12 weeks	Objective PA (StepWatch)<br><br>At baseline and 12 weeks	No between-group differences or within-group differences were observed for monitor wear time at either baseline or post intervention.<br><br>A significant intervention effect favouring the intervention group was observed for the absolute number of daily sedentary minutes after adjusting for baseline sedentary time and monitor wear time.
Cohen et al., 2017	NRCT (4 arms)	89 participants; Age, 52.8 ± 8.5 yrs; BMI, 42.6 ± 9.6 kg/m^2	Framed interactive theory-driven texting messages focused on behaviours critical to achieving and sustaining weight loss: nutrition, exercise, and self-monitoring of weight, diet, and exercise.<br>IG1: Gain-framed messages that where a match for their orientation<br>IG2: Gain-framed messages that were a mismatch for their orientation<br>IG3: Loss-framed messages that were a match<br>IG4: Loss framed messages that were a mismatch.	Problem solving<br>Feedback on behaviour<br>Self-monitoring of behaviour<br>Self-monitoring of outcomes of behaviour<br>Social support unspecified<br>Information about health consequences<br>Prompt/cues	Digital	28 days	Self-reported PA (International Physical Activity Questionnaire)<br><br>At baseline and at week 4	There were significant increases in PA in all groups.<br><br>Intervention: No significant main effects or interactions were found for IPAQ outcomes.

Authors, Year	Study Design	Sample	Intervention				Outcomes, Instruments & Time points	Physical Activity Changes
			Description	Theory & Strategies	Mode of Delivery	Length + Follow-up		
Collins et al., 2012	RCT (3 arms)	Enhanced WL (IG1): 106 participants (58% women); Age, 42.2 ± 10.2 yrs; BMI, 32.3 ± 4.3 kg/m^2<br><br>Basic WL (IG2): 99 participants (59% women); 42 ± 10.9 yrs; BMI, 32.3 ± 3.6 kg/m^2<br><br>Control (CG): 104 participants (58% women); Age, 41.7 ± 9.4 yrs; BMI, 32.2 ± 3.9 kg/m^2	Commercial Web-based weight-loss program based on social cognitive theory and targeted key mediators of behaviour change<br><br>IG1: Commercial web-based weight loss program with additional personalized e-feedback and contact from the provider (enhanced)<br><br>IG2: Standard commercial web-based weight loss program (basic)<br><br>CG: wait-list control group	Social cognitive theory<br><br>Goal setting (behaviour)<br>Goal setting outcome (IG1)<br>Feedback on behaviour (IG1)<br>Self-monitoring of behaviour<br>Self-monitoring of outcomes of behaviour<br>Feedback on outcomes of behaviour (IG1)<br>Social support (unspecified)<br>Demonstration of behaviour (IG1)<br>Prompt/cues<br>Behavioural practice/rehearsal (IG2)<br>Body changes	Digital	12 weeks	Self-reported PA (International Physical Activity Questionnaire)<br><br>Objective PA (pedometer Yamax Digi-Walker SW-700)<br><br>At baseline and 12 weeks	No significant change in total PA METs with the average step count per day decreasing in the controls but increasing in the basic and enhanced groups. There was a significantly greater increase in enhanced relative to control.
Conroy et al., 2011	RCT (3 arms)	210 participants (83.6% women); Age, 47.3 ± 8.81 yrs; BMI, 34.1 ± 4.5 kg/m^2	Behavioural intervention for weight loss involving 16 weekly and 4 biweekly group sessions during the first 6 months (nutritional and behavioural counselling). Three groups, each using a different self-monitoring tool:<br><br>IG1: PDA + FB - personal digital assistant + daily tailored feedback<br><br>IG2: PDA - personal digital assistant<br><br>IG3: PR - paper record	Goal setting (behaviour)<br>Review behaviour goals<br>Discrepancy between current behaviour and goal (IG1)<br>Feedback on behaviour<br>Self-monitoring of behaviour<br>Graded tasks	Digital	6 months + 18 months follow-up	PA adherence (min/week) recorded by participants in PR or PDA) divided by PA goals at both baseline and 6 months<br><br>Self-reported PA (Modifiable Activity Questionnaire)<br><br>At baseline and 6 months	Participants in the PDA and PDA + FB arms were more likely to demonstrate high (i.e., 100%) adherence to weekly PA goals, although the adherence in all three arms declined over time.<br><br>The participants in the PDA + FB arm demonstrated the lowest decline in PA adherence over time, and this difference was significantly lower than the declines in both the PR and the PDA arms.

Authors, Year	Study Design	Sample	Intervention				Outcomes, Instruments & Time points	Physical Activity Changes
			Description	Theory & Strategies	Mode of Delivery	Length + Follow-up		
Gill et al., 2019	RCT (2 arms)	Intervention (IG): 59 participants (76.3% women); Age, 56.8 ± 12.3 yrs; BMI, 32 ± 9.3 kg/m^2<br><br>Control (CG): 59 participants (81.4% women); Age, 58.6 ± 14.7 yrs; BMI, 30.9 ± 7.3 kg/m^2	IG: 4x 30-40 min coach sessions to set exercise, PA and healthy eating prescriptions; discuss strategies to achieve goals; personalized to participant focusing on SMART goals; with access to eHealth Tools<br><br>CG: Wait-list	Goal setting (behaviour) (IG)<br>Action planning (IG)<br>Self-monitoring of behaviour (IG)<br>Social support (unspecified) (IG)<br>Instruction on how to perform the behaviour (IG)<br>Graded tasks (IG)	Digital	6 months; Follow up at 6 months (both groups), at 12 and 18 months (interventi on only)	Self-reported PA (International Physical Activity Questionnaire)<br><br>Objective PA (Yamax Digiwalker; SW-200)<br><br>At baseline, 6, 12 and 18 months	Significant difference between average daily steps from baseline to 6 months, favouring the intervention.<br><br>When total physical activity was measured using the IPAQ, there were no differences between or within groups.
Hales et al., 2016	RCT (2 arms)	Experimental (IG): 26 participants (85% women); Age, 48.4 ± 11.9 yrs; BMI, 36.2 ± 6.3 kg/m^2<br><br>Comparison (CG): 25 participants (80% women); Age, 43.9 ± 12.7 yrs; BMI, 33.2 ± 5.3 kg/m^2	IG: Mobile app targeting social support and self-monitoring of diet, PA, and weight among overweight and obese adults<br><br>CG: Calorie counter by Fat Secret app	Social Cognitive Theory<br><br>Goal setting (behaviour) (IG)<br>Problem solving (IG)<br>Self-monitoring of behaviour<br>Social support (practical) (IG)<br>Social support (emotional) (IG)<br>Information about health consequences (IG)<br>Social comparison (IG)<br>Prompt/cues (IG)<br>Non-specific incentive (IG)<br>Incentive (outcome) (IG)<br>Reward (outcome) (IG)<br>Framing/reframing (IG)	Digital	3 months	Self-reported PA (Paffenberger Physical Activity Questionnaire)<br><br>At baseline, 3 months	There were no significant differences in final reported caloric expenditure by group assignment, with both increasing caloric expenditure during PA following the intervention.

Authors, Year	Study Design	Sample	Intervention				Outcomes, Instruments & Time points	Physical Activity Changes
			Description	Theory & Strategies	Mode of Delivery	Length + Follow-up		
Hurkmans et al., 2018	RCT (4 arms)	App (IG1): 30 participants (72% women); Age, 44 ± 12.4 yrs; BMI, 32 ± 2.1 kg/m^2<br>Combi (IG2): 22 participants (48% women); Age, 45 ± 9.6 yrs; BMI, 32 ± 2.2 kg/m^2<br>Conventional (IG3): 28 participants (84% women); Age, 46 ± 9.2 yrs; BMI, 32 ± 2 kg/m^2<br>Control group (CG): 22 participants (75% women); Age, 45 ± 10.2 yrs; BMI, 32 ± 2.0 kg/m^2	IG1: Mobile weight loss app, including digital advice for dietary pattern and PA, how to challenge themselves, self-monitoring (step count), library with (scientific) information on nutrition and PA, recipes, a help button for advice, and a link to a Facebook group<br><br>IG2: Partial face-to-face or partial app program<br><br>IG3: Received an individualized diet plan from a registered dietician and a personalized PA plan for 12 weeks from a PA coach.<br><br>CG: No intervention	Goal setting (behaviour) (IG3; IG2)<br>Problem solving (IG3; IG2)<br>Action planning (IG3; IG2)<br>Self-monitoring of behaviour	Digital	12 weeks	Self-reported PA (International Physical Activity Questionnaire)<br><br>At baseline and 12 weeks	No significant group by time effects were found for moderate and vigorous PA. Furthermore, no significant changes were found in any of the groups regarding the percentage of participants that fulfilled the IPAQ minimally active criteria and the HEPA active criteria.
Hutchesson et al., 2018	RCT (12 arms)	Intervention (IG): 29 women; Age, 26.3 ± 4.3 yrs; BMI, 29.3 ± 2.5 kg/m^2<br><br>Control (CG): 28 women; Age, 27.9 ± 5 yrs; BMI, 29.4 ± 2.5 kg/m^2	IG: program delivered using e-Health technologies only, comprising five delivery modes (website, app, email, text messages and social media) and using social cognitive theory and control theory theoretical frameworks.<br><br>CG: Waiting list	Social Cognitive Theory and Control Theory<br>Goal setting (behaviour) (IG)<br>Problem solving (IG)<br>Goal setting (outcome) (IG)<br>Discrepancy between current behaviour and goal (IG)<br>Feedback on behaviour (IG)<br>Self-monitoring of behaviour (IG)<br>Self-monitoring of outcomes of behaviour (IG)<br>Feedback on outcomes of behaviour (IG)<br>Social support (unspecified) (IG)<br>Instruction on how to perform the behaviour (IG)<br>Prompts/cues (IG)<br>Non-specific reward (IG)<br>Self-reward (IG)<br>Avoidance/reducing exposure to cues for the behaviour (IG)<br>Framing/reframing (IG)	Digital	6 months	Self-reported PA (Godin Leisure Time Exercise Questionnaire)<br><br>At baseline and 6 months	No significant group by time effects were found for moderate and vigorous PA.

Authors, Year	Study Design	Sample	Intervention				Outcomes, Instruments & Time points	Physical Activity Changes
			Description	Theory & Strategies	Mode of Delivery	Length + Follow-up		
Hochsmann, 2019	RCT (2 arms)	Intervention (IG): 18 participants (44% women); Age, 56 ± 5 yrs; BMI, 32 ± 4 kg/m^2<br><br>Control (CG): 18 participants (50% women); Age, 58 ± 6 yrs; BMI, 34 ± 5 kg/m^2	IG: PA-promoting smartphone game, whose goal is to restore a decayed garden by planting trees and flowers. The game's physical activity content includes in-game workouts as well as the promotion of daily physical activity.<br><br>CG: One lifestyle counselling aiming at increasing PA adherence	Self-determination Theory<br><br>Goal setting (behaviour) (IG)<br>Action planning (IG)<br>Feedback on behaviour (IG)<br>Instruction on how to perform the behaviour (IG)<br>Demonstration of behaviour (IG)<br>Prompt/cues (IG)<br>Behavioural practice/rehearsal<br>Graded tasks<br>Credible source (IG)<br>Non-specific reward (IG)	Digital	24 weeks	Objective PA - steps/day (Garmin Vivofit2 activity wristband); weekly total and in-game<br><br>At baseline and 24 weeks	Intervention led to stable increases in PA in inactive patients with type 2 diabetes over a 24-week period
Kurtzman et al., 2018	RCT (3 arms)	Gamification (IG1): 66 participants (83.3% women); Age, 42.3 ± 11.5 yrs; BMI, 36.0 ± 5.2 kg/m^2<br><br>Gamification + PCP (IG2): 64 participants (81.3% women); Age, 39.3.6 ± 10.6 yrs; BMI, 35.5 ± 5.0 kg/m^2<br><br>Control group: 66 participants (92.4% women); Age, 42.5 ± 12.9 yrs; BMI, 37.2 ± 5.7 kg/m^2	IG1: Smartphone app (Withings HealthMate); Entered game with a playmate and signed a commitment pledge to try their best to achieve their step goal by 24 weeks and maintain it through 36 weeks.<br><br>IG2: Same as Gamification-only group + Data sent to Primary Care Practitioner regularly over the 36 weeks.<br><br>CG: No intervention	Based on Behavioural Economics<br><br>Goal setting (behaviour)<br>Goal setting (outcome)<br>Behavioural contract (IG1; IG2)<br>Social support (unspecified) (IG1; IG2)<br>Graded tasks (IG1; IG2)<br>Material incentive (behaviour) (IG1; IG2)<br>Social incentive (IG1; IG2)<br>Behaviour cost (IG1; IG2)	Digital	24 weeks + 12 weeks follow-up	Objective PA - steps/day and proportion of participant-days achieving step goals (smartphone app Withings HealthMate)<br><br>At baseline, 24 and 36 weeks	The gamification arm had the highest mean step count, but there were no significant differences between each of the intervention arms and control.<br><br>During the 24-week intervention, the proportion of participant days where the goal of 10,000 steps was achieved was 15.5% in control, 17.0% in IG1, and 12.4% in IG2. These declined to 11.4, 12.9, and 10.3%, respectively, during the follow-up period.

Authors, Year	Study Design	Sample	Intervention				Outcomes, Instruments & Time points	Physical Activity Changes
			Description	Theory & Strategies	Mode of Delivery	Length + Follow-up		
Laing et al., 2014	RCT (2 arms)	Usual Care + App prescription (IG): 105 participants (86.5% women); Age, 47.6 ± 11.9 yrs; BMI, 37.2 ± 6.5 kg/m2<br>Usual Care (CG): 107 participants (86.5% women); Age, 47.6 ± 11.9 yrs; BMI, 37.2 ± 6.5 kg/m2	IG: Access to MyFitnessPal app, including elements of social cognitive theory<br><br>CG: No intervention	Based on Social Cognitive Theory<br><br>Goal setting (outcome) (IG)<br>Self-monitoring of behaviour (IG)<br>Self-monitoring of outcomes of behaviour (IG)<br>Feedback on outcomes of behaviour (IG)<br>Social support (unspecified) (IG)<br>Prompt/cues (IG)	Digital	6 months	Self-reported PA (survey question: "number of days doing more than 30-min PA in the last 7 days")<br><br>At baseline, 3, and 6 months.	No change in physical activity.
McConnon et al., 2007	RCT (2 arms)	Total: 221 participants (77% women); Age, 45.8 ± 10.6 yrs;<br>Internet (IG): 111 participants; BMI, 34.5 ± 3.3 kg/m^2<br>Usual Care (CG): 110 participants; BMI, 34.4 ± 3.5 kg/m^2	IG: The website provided personalized advice and motivational statements based on self-reported progress on reaching their change goals, details on progress, tools and information to support behavior change in dietary and PA patterns.<br><br>CG: No intervention	Feedback on behaviour (IG)<br>Self-monitoring of outcomes of behaviour (IG)<br>Prompt/cues (IG)<br>Non-specific incentive (IG)	Digital	12 months	Self-reported PA (Baecke physical activity questionnaire)<br><br>At baseline, 6 and 12 months	No significant differences in change in PA outcome measures between the two groups at six or 12 months were revealed.
Mensorio et al., 2019	RCT (2 arms)	Total: 106 participants, Age, 53 ± 8.9 yrs<br><br>Intervention (IG): 55 participants (85% women); 30.1 ± 2.7 kg/m^2<br><br>Control (CG): 51 participants (86% women); BMI, 30.2 ± 3.0 kg/m^2	IG: Self-administered online intervention composed of nine modules and presented via a web page aimed at progressively establishing healthy eating habits and increased levels of PA. The sessions contained psychoeducation about what a healthy lifestyle involves and learning techniques on how to achieve it on a day-to-day basis.<br>CG: Received usual medical consultations focused on reducing cardiovascular risk factors.	The program followed a cognitive-behavioral approach and was based on behavioral therapy techniques.<br><br>Problem solving (IG)<br>Self-monitoring of behaviour (IG)<br>Instruction on how to perform the behavior (IG)<br>Information about health consequences (IG)<br>Prompt/cues (IG)<br>Behavioural practice/rehearsal (IG)<br>Pros and cons (IG)<br>Self-incentive (IG)<br>Valued self-identity (IG)	Digital	3 months + 9-month follow-up	Self-reported PA (International Physical Activity Questionnaire)<br><br>At baseline, 3, 6 and 12 months	The intervention by time interaction effect on PA was significant, but main effects were not. The intervention group increased PA level at 3 months, while the control group decreased.

Authors, Year	Study Design	Sample	Intervention				Outcomes, Instruments & Time points	Physical Activity Changes
			Description	Theory & Strategies	Mode of Delivery	Length + Follow-up		
Morgan et al., 2011	RCT (2 arms)	Intervention (IG): 65 men; Age, 44.8 ± 8.3 yrs; BMI, 30.7 ± 3.6 kg/m^2<br><br>Control (CG): 45 men; Age, 43.7 ± 9.1 yrs; BMI, 30.2 ± 3.5 kg/m^2	IG: 1 face-to-face session covering education about energy balance, the challenges of shift work relating to diet and PA, weight loss tips for men, and behavior change strategies + a weight loss handbook, a website user guide and a YamaxSW200 pedometer.<br><br>CG: Waiting list	Social Cognitive Theory<br><br>Goal setting (behaviour) (IG)<br>Action planning (IG)<br>Feedback on behaviour (IG)<br>Self-monitoring of behaviour (IG)<br>Self-monitoring of outcomes of behaviour (IG)<br>Social support (unspecified) (IG)<br>Information about health consequences (IG)<br>Credible source (IG)<br>Material incentive (behaviour) (IG)<br>Material reward (IG)<br>Social reward (IG)<br>Verbal persuasion about capability (IG)	Digital	3 months	Self-reported PA (Godin Leisure Time Exercise Questionnaire)<br><br>At baseline and 3 months	Significant changes in PA favouring the intervention group in Total MET minutes and leisure time PA.
Napolitano et al., 2013	RCT (3 arms)	Total: 52 participants; Age, 20.5 ± 2.2 yrs; BMI, 31.4 ± 5.3 kg/m^2<br><br>Facebook (IG1): 17 participants<br><br>Facebook Plus condition (IG2): 18 participants<br><br>Control (CG): 17 participants	IG1: Participants received handouts and podcasts on healthy nutrition and PA and were alerted to the availability of new intervention content via group postings and Facebook mail. Participants were encouraged to gradually increase their PA with the target of engaging in moderate intensity exercise for at least 250 minutes per week<br><br>IG2: Participants received additional theoretically driven intervention targets: goal setting, individualized feedback on self-monitoring and progress, encouragement<br><br>CG: Waiting list	Based on self-regulation theory<br><br>Goal setting (behaviour) (IG1; IG2)<br>Problem solving (IG2)<br>Goal setting (outcome) (IG2)<br>Review behaviour goals (IG2)<br>Feedback on behaviour (IG2)<br>Self-monitoring of behaviour (IG2)<br>Feedback on outcomes of behaviour (IG2)<br>Social support (unspecified) (IG2)<br>Social comparison (IG1)<br>Graded tasks (IG1)<br>Social reward (IG1; IG2)	Digital	8 weeks	Self-reported PA (Godin Leisure Time Exercise Questionnaire)<br><br>At baseline and 8 weeks	No significant changes in physical activity.

Authors, Year	Study Design	Sample	Intervention				Outcomes, Instruments & Time points	Physical Activity Changes
			Description	Theory & Strategies	Mode of Delivery	Length + Follow-up		
Patrick et al., 2011	RCT (2 arms)	Intervention (IG): 224 men; Age, 44.9 ± 7.8 yrs; BMI, 34.2 ± 4.2 kg/m^2<br><br>Control (CG): 217 men; Age, 42.8 ± 8 yrs; BMI, 34.3 ± 4 kg/m^2	IG: Intervention focused on making small, incremental improvements over time. The web site included skill-building tools and PA and nutrition information Participants had an opportunity to e-mail a question to our study experts.<br><br>CG: Waiting list	Based on social cognitive theory and behavioral (exercise) determinants model<br><br>Goal setting (behaviour) (IG)<br>Feedback on behaviour (IG)<br>Self-monitoring of behaviour (IG)<br>Social support (unspecified) (IG)<br>Instruction on how to perform the behaviour (IG)<br>Prompt/cues (IG)<br>Graded tasks (IG)	Digital	12 months	Self-reported PA (International Physical Activity Questionnaire)<br><br>At baseline, 6 and 12 months	Significant group-by-time interactions were found for estimated total walking minutes per day for 6 and 12 months, with men in the intervention group walking about 16 minutes/day more than men in the control group at 12 months.
Richardson et al., 2010	RCT (2 arms)	Intervention (IG): 254 participants (64% women); Age, 51.7 ± 11.3 yrs; BMI, 33.1 ± 6.3 kg/m^2<br><br>Control (CG): 70 participants (66% women); Age, 53.3 ± 11.8 yrs; BMI, 33.4 ± 5.8 kg/m^2	IG: Internet-mediated walking program with pedometer and access to online community features embedded in their intervention webpage<br><br>CG: Participants could not read or post messages to other control-arm participants	Bandura's social-cognitive theory and social influence theories including social learning theory<br><br>Goal setting (behaviour) (IG)<br>Feedback on behaviour (IG)<br>Self-monitoring of behaviour (IG)<br>Social support (unspecified) (IG)<br>Demonstration of behaviour (IG)<br>Social comparison (IG)<br>Graded tasks (IG)<br>Material reward (behaviour) (IG)<br>Social reward (IG)<br>Reward approximation (IG)<br>Vicarious consequences (IG)	Digital	4 months	Objective PA - step counts (Omron HJ-720-ITC pedometer).<br><br>At baseline and 4 months	Both arms significantly increased their average daily steps between baseline and the end of the intervention period (~1 mile increase), and there were no significant differences between arms.
Shapiro et al., 2012	RCT (2 arms)	Intervention (IG): 81 participants (67% women); Age, 43.1 ± 11.4 yrs; BMI, 32.4 ± 4.2 kg/m^2<br><br>Control (CG): 89 participants (64% women); Age, 40.9 ± 12.1 yrs; BMI, 32.0 ± 4.0 kg/m^2	IG: Participants received SMS and MMS 4 times/day for 12 months. SMS included: tips, facts, motivation, messages requesting answers to knowledge questions, or self-monitoring data on weight and steps. MMS included portion control pictures and weight/step graphical feedback over time.<br>CG: Received the same monthly e-newsletters as the IG but did not receive SMS, MMS, or have access to the intervention website.	Social Cognitive Theory<br><br>Goal setting (behaviour) (IG)<br>Review behaviour goals (IG)<br>Feedback on behaviour (IG)<br>Self-monitoring of behaviour (IG)<br>Self-monitoring of outcomes of behaviour (IG)<br>Feedback on outcomes of behaviour (IG)<br>Prompt/cues (IG)<br>Graded tasks (IG)<br>Credible source (IG)<br>Social reward (IG)	Digital	12 months	Objective PA - daily step counts (Yamax Digi-Walker CW-Series 600 pedometer).<br><br>At baseline, 6 and 12 months	Among completers, steps continued to increase throughout the 12-month study period. In intent-to treat analysis, there was a linear increase in steps over the first 6 months that leveled off thereafter.

Authors, Year	Study Design	Sample	Intervention				Outcomes, Instruments & Time points	Physical Activity Changes
			Description	Theory & Strategies	Mode of Delivery	Length + Follow-up		
Sniehotta et al., 2019	RCT (2 arms)	Intervention (IG): 144 participants (76.4% women); Age, 42.0 ± 11.6 yrs; BMI, 30.9 ± 5.5 kg/m^2<br><br>Control (CG): 144 (78.5% women); Age, 41.6 ± 11.4 yrs; BMI, 30.8 ± 5.2 kg/m^2	IG: Combination of a single face-to-face meeting with an intervention team member and regular automated SMS with embedded links and other content, along with personalized SMS generated by the intervention team. Participants were prompted to log on to an online study interface regularly.<br><br>CG: Participants received standard lifestyle advice delivered in SMS with embedded links. Content from the NHS Choices website.	Self-regulation theory, health action process approach<br><br>Goal setting (behaviour) (IG)<br>Problem solving (IG)<br>Goal setting (outcome) (IG)<br>Feedback on behaviour (IG)<br>Self-monitoring of behaviour (IG)<br>Social support (unspecified) (IG)<br>Prompt/cues (IG)<br>Social reward (IG)	Digital	12 months	Objective PA (ActiGraph GT3X+)<br><br>At baseline and 12 months.	Both groups became less physically active over the study. There was a small but significant difference between the arms such that intervention group participants were more physically active at 12-month follow-up than control participants, adjusting for baseline levels of PA.
Steinberg et al., 2013	RCT (2 arms)	Intervention (IG): 47 participants (70% women); Age, 43.0 ± 11.4 yrs; BMI, 33.2 ± 4 kg/m^2<br><br>Control (CG): 44 participants (80% women); Age, 44.7 ± 10.6 yrs; BMI, 31.1 ± 3.1kg/m^2	IG: Consisted of 4 main components: 1) cellular connected ‘‘smart’’ scale for daily weighing; 2) web-based graph of weight trends over time; 3) weekly tailored feedback via e-mail on self-weighing frequency and weight loss progress; and 4) 22 weekly lessons on behavioral weight control via e-mail. Lessons were both informational and behavioral, and included topics such as portion control, restaurant eating, structured exercise.<br><br>CG: Delayed intervention control group was instructed to maintain their current self-weighing habits.	Goal setting (behaviour) (IG)<br>Problem solving (IG)<br>Feedback on behaviour (IG)<br>Self-monitoring of outcomes of behaviour (IG)<br>Feedback on outcomes of behaviour (IG)<br>Instruction on how to perform the behaviour (IG)<br>Demonstration of behaviour (IG)	Digital	6 months	Self-reported PA (Paffenbarger Exercise Habits Questionnaire)<br><br>Baseline, 3, and 6 months.	There were no differences between groups with regard to calories expended per week from PA, although there was a trend toward greater PA over time among the intervention group, with the difference almost reaching statistical significance at 3 months.

Authors, Year	Study Design	Sample	Intervention				Outcomes, Instruments & Time points	Physical Activity Changes
			Description	Theory & Strategies	Mode of Delivery	Length + Follow-up		
Stephens et al., 2017	RCT (2 arms)	Intervention (IG): 31 participants (70% women); Median age 20 yrs; median BMI 29 kg/m^2<br><br>Control (CG): 31 participants (80% women); Median age 20 yrs; Median BMI 26.6 kg/m^2	IG: Participants were guided to download and use the Lose it! Application to self-monitor behavior.<br>The application offered social networking through a "friend" feature. Participants were encouraged to identify specific goals that their health coach could help them achieve. Individualized text messages were delivered to the participants' smartphone from a health coach at frequency determined by the participant.<br>CG: Participants were asked not to use any Smartphone applications focused on weight loss for the duration of the study	Self-efficacy theory, social cognitive theory.<br><br>Goal setting (behaviour) (IG)<br>Feedback on behaviour (IG)<br>Self-monitoring of behaviour (IG)<br>Social support (unspecified) IG)	Digital	3 months	Self-reported PA (Godin Leisure Time Exercise Questionnaire).<br><br>At baseline and 3 months.	The intervention group improved overall PA performed, but the differences were not significant when compared to the control group.
Tate et al., 2003	RCT (2 arms)	Basic internet (IG1): 46 participants (89% women); Age, 47.3 ± 9.5 yrs; BMI, 33.7 ± 3.7 kg/m^2<br><br>Internet behavioral e-counseling (IG2): 46 participants (91% women); Age, 49.8 ± 9.3 yrs; BMI, 32.5 ± 3.8 kg/m^2	IG1: Web site provided a tutorial on weight loss, a new tip and link each week, and a directory of selected Internet resources. Each week, participants received an e-mail reminder to submit his/her weight and received weight loss information.<br>IG2: Besides the basic internet, participants communicated with their weight loss counselor. During the first month, the therapist emailed participants 5 times per week. Therapists sent weekly e-mails for the remaining 11 months.<br><br>Both groups received an introductory session with instructions on diet, PA, and behavior change. Energy restriction and expenditure targets were recommended	Goal setting (behaviour)<br>Feedback on behaviour (IG2)<br>Self-monitoring of behaviour<br>Social support (unspecified) (IG2)<br>Instruction on how to perform the behaviour<br>Prompt/cues<br>Social reward (IG2)	Digital	12 months	Self-reported PA (Paffenbarger Exercise Habits Questionnaire)<br><br>At baseline and 12 months.	There were no significant differences between and within groups.

Authors, Year	Study Design	Sample	Intervention				Outcomes, Instruments & Time points	Physical Activity Changes
			Description	Theory & Strategies	Mode of Delivery	Length + Follow-up		
Tate et al., 2006	RCT (3 arms)	E-counseling + tailored automated feedback (IG1): 61 participants (87% women); Age, 49.7 ± 11.4 yrs; BMI, 32.7 ± 3.5 kg/m^2<br><br>E-counseling + Human Counselor (IG2): 64 participants (84% women); Age, 47.9 ± 9.8 yrs; BMI, 32.8 ± 3.4 kg/m^2<br><br>No counseling (CG): 67 participants (82% women); Age, 49.9 ± 8.3 yrs; BMI, 32.3 ± 3.7 kg/m^2	All participants attended one face-to-face group session with instructions on diet, exercise, and behavior change, and were instructed on how to use the Slim-Fast Web site, including weekly reporting, progress graphs, weight loss tips.<br><br>IG1 and IG2: Access to a Web site that offered additional features, including an electronic diary and a message board.<br><br>IG1: Tailored automated feedback through an algorithm.<br><br>IG2: Tailored feedback provided by counselor via e-mail.<br><br>CG: Only the face-to-face group session.	Goal setting (behaviour)<br>Problem solving (IG1; IG2)<br>Discrepancy between current behaviour and goal (IG1; IG2)<br>Feedback on behaviour (IG1; IG2)<br>Self-monitoring of behaviour<br>Self-monitoring of outcomes of behaviour<br>Feedback on outcomes of behaviour<br>Social support (unspecified)<br>Social comparison<br>Prompts/cues<br>Social reward (IG1; IG2)	Digital	6 months	Self-reported PA (Paffenbarger Exercise Habits Questionnaire)<br><br>At baseline, 3 and 6 months.	No treatment by time interaction; however, all groups showed increased PA during the first 3 months, which subsequently declined between 3 and 6 months
Turner-McGrievy et al., 2011	RCT (2 arms)	Podcast+mobile (IG1): 47 participants (77% women); 42.6 ± 10.7; BMI, 32.9 ± 4.8 kg/m^2<br><br>Podcast (IG2): 49 participants (73% women); Age, 43.2 ± 11.7 yrs; BMI, 32.2 ± 4.5 kg/m^2	IG1: Enhanced mobile approach to help people lose weight in the Mobile Pounds Off Digitally. It included nutrition and physical activity information, testimonies, soap opera, goal setting activity, overcoming barriers, problem solving<br><br>IG2: A currently available podcast that is not theory based.	Podcasts' design using constructs from social cognitive theory<br><br>Goal setting (behaviour)<br>Problem solving<br>Self-monitoring of behaviour<br>Social support (unspecified) (IG1)<br>Instruction on how to perform the behaviour<br>Information about health consequences<br>Demonstration of behaviour<br>Social comparison<br>Prompts/cues	Digital	6 months	Self-reported PA (Paffenbarger Physical Activity Questionnaire)<br><br>At baseline, 3 and 6 months	There were no significant differences between groups in energy expenditure or group-by-time interaction at 3 or 6 months

Authors, Year	Study Design	Sample	Intervention				Outcomes, Instruments & Time points	Physical Activity Changes
			Description	Theory & Strategies	Mode of Delivery	Length + Follow-up		
Turner-McGrievy et al., 2009	RCT (2 arms)	Enhanced group (IG): 41 participants (68% women); Age, 37.7 ± 11.8 yrs; BMI, 31.8 ± 3.2 kg/m^2<br><br>Control (CG): 37 participants (81% women); Age, 39.6 ± (12.2) yrs; BMI, 31.4 ± 4.1 kg/m^2	IG: Weight-loss podcast designed with health behaviour theories to support weight loss<br><br>CG: A currently available podcast that is not theory based.	The enhanced podcast was designed using constructs from Social Cognitive Theory<br><br>Goal setting (IG)<br>Self-monitoring of behaviour (IG)<br>Instruction on how to perform the behaviour (IG)<br>Information about health consequences (IG)<br>Demonstration of behaviour (IG)	Digital	12 weeks	Self-reported PA (International Physical Activity Questionnaire)<br><br>At baseline and 12 weeks	There was no significant difference between groups with regard to reported moderate activity or walking. There was a difference in reported vigorous activity, with a greater increase in the number of days participants engaged in vigorous activity seen in the enhanced group.
van Genugten et al., 2012	RCT (2 arms)	Computer tailored (IG): 269 participants (16% women); Age, 47.7 ± 9.2 yrs; BMI, 28.17 ± 2.02 kg/m^2<br><br>Generic information website (CG): 270 participants (20% women); Age, 47.9 ± 9.7; BMI, 27.91 ± 1.85 kg/m^2	IG: Computer-tailored intervention, consisting of 4 modules, each to be visited 1 week after the other. Completion of the entire program took about 90 min in total. The first module aimed at commitment to prevent weight gain, the second and third modules were focused on evaluation of progress toward behaviour change, and the fourth on promoting sustained changes.<br><br>CG: Generic information	Theory and evidence using the Intervention Mapping: Self-Regulation Theory, Theory of Planned Behaviour, Precaution Adoption Process Model and Implementation Intentions.<br><br>Problem solving (IG)<br>Action planning (IG)<br>Review of behaviour goals (IG)<br>Behavioural contract (IG)<br>Feedback on behaviour<br>Self-monitoring of behaviour (IG)<br>Social support (unspecified) (IG)<br>Instruction on how to perform the behaviour<br>Information about health consequences (IG)<br>Demonstration of behaviour (IG)<br>Prompt/cues<br>Behavioural practice/rehearsal (IG)<br>Pros and cons (IG)<br>Social reward (IG)	Digital	1 month + 6 months follow-up	Self-reported PA (Short Questionnaire to Assess Health enhancing physical activity)<br><br>Baseline, 1 and 6 months	The time spent on physical activity decreased significantly in the total population, but the change was not significantly different among the tailored intervention and generic information conditions.

Authors, Year	Study Design	Sample	Intervention				Outcomes, Instruments & Time points	Physical Activity Changes
			Description	Theory & Strategies	Mode of Delivery	Length + Follow-up		
van Wier et al., 2009	RCT (3 arms)	Phone condition (IG1): 462 participants (30.5% women); Age, 43 ± 8.8 yrs; BMI, 29.5 ± 3.5 kg/m^2<br><br>Internet condition (IG2): 464 participants (34.9% female); Age, 43 ± 8.4 yrs; BMI, 29.6 ± 3.4 kg/m^2<br><br>Control (CG): 460 participants (33.5% female); Age, 43 ± 8.7 yrs, BMI, 29.6 ± 3.7 kg/m^2	To evaluate, among an overweight working population, the effectiveness of a lifestyle intervention programme on bodyweight, physical activity and eating habits.<br><br>IG1: Intervention content was provided in a binder. Counselling sessions every two weeks by appointment. Feedback on module completion by phone.<br><br>IG2: Access to interactive website with web pages containing general information. Feedback on module completion by email.<br><br>CG: Self-help materials only. No counseling.	Social cognitive theory<br><br>Goal setting (behaviour) (IG1; IG2)<br>Feedback on behaviour (IG2)<br>Self-monitoring of behaviour (IG1; IG2)<br>Instruction on how to perform the behaviour (IG1; IG2)<br>Demonstration of behaviour (IG1; IG2)<br>Prompts/cues (IG2)<br>Behavioural practice/rehearsal (IG1; IG2)<br>Credible source (IG1; IG2)<br>Avoidance/reducing exposure to cues for the behaviour	Digital	6 months + 2 years follow up	Self-reported PA (Short Questionnaire to Assess Health enhancing physical activity)<br><br>At baseline and 6 months	The comparison of the phone group with the control group showed statistically significant changes in PA. No other differences were found.
Wang et al., 2015	RCT (2 arms)	Intervention (IG): 33 participants (88% women); 49.3 ± 11.5 yrs; BMI > 25<br><br>Active Control (CG): 34 participants (94% women); 47.1 ± 11.9 yrs; BMI > 25	To set the PA agenda for all participants, study personnel provided a brief 5-min intervention to review motivation, set goals (i.e., toward 10,000 steps/day), and plan for challenging situations.<br><br>IG: Technology-based intervention that delivered simple prompts using SMS text messaging in conjunction with the Fitbit One for self-monitoring<br><br>CG: Self-monitoring with Fitbit One only.	Goal setting (behaviour)<br>Problem solving<br>Self-monitoring of behaviour<br>Prompt/cues (IG)<br>Credible source	Digital	6 weeks	Objective PA - number of steps and minutes of PA<br><br>Actigraph (Pen-sacola, FL) GT3X+ (primary measure) and Fitbit One (secondary measure)<br><br>At baseline and weeks 1, 2, 3, 4, 5, and 6	The intervention group showed on average higher activity levels over the 6 weeks compared with the comparison group.<br><br>There were no between-group differences in changes for steps or minutes of PA by intensity level.

Authors, Year	Study Design	Sample	Intervention				Outcomes, Instruments & Time points	Physical Activity Changes
			Description	Theory & Strategies	Mode of Delivery	Length + Follow-up		
Watson et al., 2012	RCT (2 arms)	Intervention (IG): 35 participants (89% women); Age, 44.1 ± 9 median yrs; BMI, 30.2 ± 30.1 median kg/m^2<br>Control (CG): 35 participants (80% women); Age, 40.6 ± 41 median yrs; BMI, 30.4 ± 29.7 median kg/m^2	IG: Virtual coaching, pedometer, and website. Program focused on building rapport and establishing baseline activity levels, followed by tips to increase activity, daily personalized goal setting, and advice about maintaining a healthy diet and activity level after the study concludes.<br>CG: Use of a pedometer and website alone	Goal setting (behaviour)<br>Problem solving (IG)<br>Goal setting (outcome) (IG)<br>Review behaviour goals (IG)<br>Commitment (IG)<br>Feedback on behaviour (IG)<br>Self-monitoring of behaviour<br>Social support (unspecified) (IG)<br>Instruction on how to perform the behaviour (IG)<br>Social reward (IG)	Digital	12 weeks	Objective PA - step count (Pedometer)<br>Self-reported PA (7-day physical activity recall)<br>At baseline, 3, 6, 9 and 12 weeks.	The average step count in the control group fell significantly. The intervention participants' mean step count did not change significantly. The difference seen between groups between baseline and week 9 was statistically significant.<br>Percentage change in step count across all study periods was significantly different in the intervention versus control arms.
Watson et al., 2015	RCT (2 arms)	Intervention (IG): 32 participants (50% women), Age, 51.4 ± 7.59 yrs; BMI, 32.9 ± 3.07 kg/m^2<br>Control (CG): 33 participants (61% female); Age, 52.9 ± 7.27 yrs; BMI, 32.4 ± 2.74 kg/m^2	IG: Interactive Web-based component to assist in lifestyle change, focusing on improving diet, increasing PA, and managing weight. It combines objective monitoring of weight and physical activity with automated, tailored feedback and support by physiologists by telephone and email.<br>CG group: No intervention	Goal setting (behaviour) (IG)<br>Problem solving (IG)<br>Action planning (IG)<br>Review behaviour goals (IG)<br>Feedback on behaviour (IG)<br>Self-monitoring of behaviour (IG)<br>Self-monitoring of outcomes of behaviour (IG)<br>Social support (unspecified) (IG)<br>Prompts/cues (IG)	Digital	3 months + 9-month follow up	Self-reported PA (Recent Physical Activity Questionnaire)<br>At baseline, 3, 6, 12 months	The intervention group significantly increased the time spent exercising at intensity greater than 3.5 METs relative to the control group from baseline to 3 months. This was not sustained over the longer term at 6 and 12 months.
West et al., 2016	RCT (2 arms)	Intervention (IG): 199 participants (89.5% women); Age, 47.9 ± 9.5; BMI, 35.9 ± 6<br>Control (CG): 199 participants (90% women); Age, 48.9 ± 10.7 yrs; BMI, 36.1 ± 6.1	IG: Behavioural treatment + Motivational Interviewing chat. Same online group program as control augmented with six individual, online motivational interviewing chat, sessions, focused on changing dietary and physical activity patterns using self-management skills and behavioural strategies.<br>CG: Group-based online, goal-directed intervention	Goal setting (behaviour)<br>Problem solving<br>Review behaviour goals (IG)<br>Discrepancy between current behaviour and goal (IG)<br>Feedback on behaviour<br>Self-monitoring of behaviour<br>Self-monitoring of outcomes of behaviour<br>Social support (unspecified) (IG)<br>Prompts/cues (IG)<br>Graded tasks<br>Credible source (IG)<br>Comparative imagining of future outcomes (IG)<br>Social rewards (IG)<br>Focus on past success (IG)	Digital	18 months	Objective PA (activity band)<br>At baseline, 6 and 18 months.	Physical activity minutes/day: no significant differences between groups

Authors, Year	Study Design	Sample	Intervention				Outcomes, Instruments & Time points	Physical Activity Changes
			Description	Theory & Strategies	Mode of Delivery	Length + Follow-up		
Akers et al., 2012	NRCT (2 arms)	Total: 40 participants (55% women); Age, 62.7 ± 0.9 yrs; BMI, 29.2 ± 1.1 kg/m^2<br>WEV (IG1): 21 participants; BMI, 29.0 ± 1.3 kg/m^2<br>WEV+ (IG2): 19 participants; BMI, 29.1 ± 0.8 kg/m^2	IG1: weight loss maintenance intervention using daily self-monitoring of body weight, step count, F/V intake<br><br>IG2: In addition, WEV+ subjects were instructed to record daily water consumption.	Goal setting (behaviour)<br>Goal setting (outcome)<br>Feedback on behaviour<br>Self-monitoring of behaviour<br>Self-monitoring of outcomes of behaviour<br>Feedback on outcomes of behaviour	Face-to-Face	12 months	Objective PA - Steps/day (pedometer; ACCUSPLIT Eagle AX120, San Jose, CA)<br><br>At baseline, 6 and 12 months	Both groups decreased steps/day at 6 and 12 months, but IG2 showed greater reductions.
Ash et al., 2006	RCT (3 arms)	FBI (IG1): 57 participants; Age, 49 ± 13 yrs; BMI, 33.7 ± 4.6 kg/m^2<br><br>IDT (IG2): 65 participants; Age, 48 ± 13yrs; BMI, 34.2 ± 5.9 kg/m^2<br><br>BO (Control): 54 participants; Age, 47 ± 14 yrs; BMI 35.8 ± 6.2 kg/m^2	IG1: Booklet + Group intervention including knowledge and skill development, cognitive behaviour therapy and relapse prevention to improve self-concept, self-efficacy, and skills mastery. Used non-directive approach; Information was available about diet and PA, but it was up to individuals if they acted on this information making changes to their lifestyle.<br>IG2: Booklet + Individualized weekly contact with dietitian for 8 weeks; nutrition assessment, provision of individualised diet prescription (aiming weight loss of 0.5-1kg/week), and an exercise prescription (20–30 min of accumulated exercise most days of the week)<br>CG: Nutrition resource booklet based on cognitive behaviour therapy principles.	Goal setting (behaviour) (IG2)<br>Problem solving (IG1)<br>Goal setting (outcome) (IG2)<br>Instruction on how to perform the behaviour<br>Behavioural practice/rehearsal (IG1)	Face-to-face	12 months	Self-reported PA (International Physical Activity Questionnaire)<br><br>At baseline, 3, 6 and 12 months	Relative to IG1, the odds of being sufficiently active at 3 months compared to baseline were 0.27 times lower in IG2 and 0.19 times lower in the CG.

Authors, Year	Study Design	Sample	Intervention				Outcomes, Instruments & Time points	Physical Activity Changes
			Description	Theory & Strategies	Mode of Delivery	Length + Follow-up		
Biddle et al., 2015	RCT (2 arms)	Intervention (IG): 94 participants; 32.4 ± 5.4 years; BMI, 34.6 ± 4.9 kg/m^2<br><br>Control (CG): 92 participants; Age, 33.3 ± 5.8 yrs; BMI, 34.5 ± 5.0 kg/m^2	IG: Education workshop focused on fostering knowledge and perceptions of prevalent risk factors for type 2 Diabetes and promoting change in sedentary behaviour. Follow-up phone call to review progress, discuss goals, and discuss barriers.<br><br>CG: Information leaflet about PA, sedentary behaviour and diabetes	Goal setting (behaviour) (IG)<br>Problem solving (IG)<br>Action planning (IG)<br>Feedback on behaviour (IG)<br>Self-monitoring of behaviour (IG)<br>Instruction on how to perform the behaviour (IG)<br>Information about health consequences<br>Demonstration of behaviour (IG)<br>Prompts/cues (IG)<br>Behavioural practice/rehearsal (IG)	Face-to-face	12 months	Objective PA (Activpal)<br><br>Self-reported PA (International Physical Activity Questionnaire)<br><br>At baseline, 3 and 12 months	No significant changes at 12 months for objective or self-reported sedentary behaviour and PA variables.
Boiché et al., 2018	NRCT (2 arms)	Intervention (IG): 24 participants (75% women); Age, 49.5 ± 13.5 yrs; BMI, 37.4 ± 5 kg/m^2<br><br>Control (CG): 25 participants (72% women); Age, 54.4 ± 9.5 yrs; BMI, 39 ± 4.6 kg/m^2	IG: SDT-based intervention in addition to a residential program. Focus on creating a motivational climate and support need. It included a PA session and a motivational session to consider experience and feelings of patients regarding PA; and discuss perspectives of PA after intervention.<br><br>CG: Standard care; walking sessions every morning; 50-minute sessions of physical rehabilitation every day	Self-determination Theory<br><br>Goal setting (behaviour) (IG)<br>Problem solving (IG)<br>Action planning (IG)<br>Feedback on behaviour (IG)<br>Social support (unspecified) (IG)<br>Behavioural practice/rehearsal<br>Graded tasks (IG)<br>Self-reward (IG)	Face-to-face	3 weeks + 1 month follow-up	Self-reported PA (validated physical activity questionnaire for the elderly)<br><br>At baseline, 3 weeks and 1 month	Increase was significantly higher among patients in the IG compared to those in the CG

Authors, Year	Study Design	Sample	Intervention				Outcomes, Instruments & Time points	Physical Activity Changes
			Description	Theory & Strategies	Mode of Delivery	Length + Follow-up		
Conroy et al., 2015	RCT (2 arms)	Interventionist Led (IG): 49 women; Age, 53.8 ± 5.3 yrs; BMI, 36.1 ± 6.0 kg/m^2<br><br>Self-Guided (CG): 49 women; Age, 54 ± 5.6 yrs; BMI, 33.4 ± 5.4 kg/m^2	IG: 12 weekly group sessions (PA, diet, and stress relief adapted) with 30 min discussions followed by 30 min of group-based, moderate-intensity PA; mindfulness practices and pedometer, calorie counter book, pedometer and sheets for tracking diet and PA.<br>CG: Self-guided manual on the American Heart Association's choose to move program, calorie counter book and pedometer	Goal setting (behaviour) (IG)<br>Self-monitoring of behaviour<br>Behavioural practice/rehearsal (IG)<br>Distraction (IG)	Face-to-face	12 wks + 9 months follow up	Self-reported PA (Modifiable Activity Questionnaire)<br><br>METs-hour/week<br><br>At baseline, 3 and 12 months	At 3 months, the IG increased PA significantly more than the CG.<br>At 12 months, there were no significant group differences.
Eaton et al., 2016	RCT (2 arms)	Enhanced Intervention (IG): 106 participants (75.2% women); Age, 48.5 ± 11.9 yrs; BMI, 37.7 ± 6.5 kg/m^2<br><br>Standard interventions (CG): 105 participants (83% women); Age, 48.6 ± 18.7 yrs; BMI, 37.8 ± 6.7 kg/m^2	IG: Tailored lifestyle intervention. Participants received phone calls from the lifestyle counsellor monthly for the first 6 months and bi-monthly for the next 6. They also received weekly mailings, print materials, feedback on food and exercise logs, and 2 exercise-related DVDs. The mailings focused on participant's motivation, weight loss, calorie and exercise goal attainment, journal compliance, food-related issues, and comorbid conditions.<br><br>CG: Participants received 5 pamphlets produced by the National Institute for Diabetes and Digestive and Kidney Diseases on weight loss, PA, and healthy eating.	Goal setting (behaviour)<br>Problem solving (IG)<br>Goal setting (outcome)<br>Action planning<br>Review behaviour goals<br>Discrepancy between current behaviour and goal<br>Feedback on behaviour (IG)<br>Self-monitoring of behaviour<br>Feedback on outcomes of behaviour<br>Social support (unspecified)<br>Instruction on how to perform the behaviour<br>Information about health consequences<br>Demonstration of behaviour (IG)<br>Social comparison (IG)<br>Information about others approval (IG)<br>Behavioural practice/rehearsal (IG)<br>Graded tasks<br>Credible source (CG)<br>Pros and cons (IG)<br>Social reward<br>Self-reward (IG)<br>Identification of self as role model (IG)<br>Identity associated with changed behaviour (IG)<br>Focus on past success (IG)<br>Self-talk (IG)	Face-to-face	12 months + 12 maintenance of higher level of PA	Self-reported PA (7-day Physical Activity Recall Questionnaire)<br><br>At baseline, 6, 12, 18 and 24 months	IG reported significantly more minutes of vigorous PA over time.<br><br>Difference between groups reached significance at 12 and 18 months.

Authors, Year	Study Design	Sample	Intervention				Outcomes, Instruments & Time points	Physical Activity Changes
			Description	Theory & Strategies	Mode of Delivery	Length + Follow-up		
Greaney et al., 2017	RCT (2 arms)	Intervention (IG): 60 participants; Age, 36.6 ± 5.1 yrs; BMI, 30.2 ± 2.8 kg/m^2<br><br>Usual care (CG): 61 participants; Age, 35.6 ± 5.8 yrs; BMI, 30.3 ± 2.4 kg/m^2	IG: Participants were provided with individual self-monitored concise and easy to comprehend tailored behaviour change goals, skills training materials, weekly telephone calls for self-monitoring behaviours, monthly telephone coaching calls with motivational interviewing principles, and a free 12 month access to YMCA facility.<br><br>CG: Mailed semi-annual newsletters during the intervention period, covering general wellness topics except physical activity, nutrition, or weight.	Social cognitive theory<br><br>Goal setting (behaviour) (IG)<br>Problem solving (IG)<br>Review behaviour goals (IG)<br>Social support (emotional) (IG)<br>Graded tasks (IG)<br>Restructuring the social environment (IG)<br>Focus on past success (IG)	Face-to-face	12 month	Objective PA (Accelerometers Actical, Philips Respiro-nics, Inc., Be)<br><br>At baseline and 12 months	No significant differences in MVPA in either group at 12 months, or in the percent of participants meeting the PA recommendation at either baseline or 12 months.
Gohner et al., 2012	NRCT (2 arms)	Intervention group (IG): 403 participants (77.5% women); Age, 48.9 ± 10.9 yrs; BMI, 35.2 ± 2.9 kg/m^2<br><br>Control group (CG): 285 participants (75.4% women); Age, 53.2 ± 10 yrs; BMI, 34.1 ± 3.2 kg/m^2	IG: Intervention with four components: medical examinations; exercise program; dietary advice; and group sessions, which consist of motivational and volitional behaviour change strategies. Motivational strategies aim at the creation of strong and self-concordant goal intentions. Volitional strategies targeted implementation skills and action control abilities.<br><br>CG: No intervention.	Goal setting (behaviour) (IG)<br>Problem solving (IG)<br>Action planning (IG)<br>Self-monitoring of behaviour (IG)<br>Pros and cons (IG)	Face-to-face	1-year program + 12-month follow-up	Self-reported PA (Short version of the Freiburg Questionnaire on Physical Exercise)<br><br>At baseline, 6, 12 and 24 months	Intervention had a substantial effect on level of physical exercise.<br><br>Results revealed significant group differences at 6, 12, and 24 even controlling for baseline values.

Authors, Year	Study Design	Sample	Intervention				Outcomes, Instruments & Time points	Physical Activity Changes
			Description	Theory & Strategies	Mode of Delivery	Length + Follow-up		
Jakicic, 2012	RCT (2 arms)	SBWI (CG): 165 participants (82% women); Age 42.4 ± 9.2 yrs; BMI, 33 ± 3.6 kg/m^2<br><br>STEP (IG1): 198 participants (83% women); Age, 42 ± 8.9 yrs; BMI, 32.9 ± 3.7 kg/m^2	CG (active control): Participants received group-based intervention sessions throughout the 18-month intervention focused on improving knowledge related to adoption and maintenance of eating and activity behaviours to promote weight loss, and strategies to facilitate long-term behavioural change<br><br>IG1: Similar to the CG, but contact frequency, contact type, and other weight loss strategies were modified depending on the achievement of specific weight loss goals at 3-month intervals (stepped care approach)	Goal setting (behaviour)<br>Problem solving<br>Goal setting (outcome) (IG1)<br>Feedback on behaviour<br>Self-monitoring of behaviour<br>Instruction on how to perform the behaviour<br>Non-specific reward	Face-to-face	18 months	Self-reported PA (Paffenbarger Physical Activity Questionnaire)<br><br>Objective PA (SenseWear ProArmband, BodyMedia Inc)<br><br>At baseline, 3, 6, 9,12, 15, and 18 months.	No statistcal significance between and within changes in PA.
Jakicic, 2015	RCT (3 arms)	IG1: 69 participants (80% women); Age, 42 ± 9.3 yrs; BMI 32.7 ± 3.7 kg/m^2<br><br>IG2: 64 participants (77% women); Age, 43.3 ± 8.6 yrs; BMI, 33.3 ± 2.9 kg/m^2<br><br>IG3: 62 participants (77% women); Age, 44.5 ± 7.6 yrs; 33.1 ± 3.6 kg/m^2	IG1: Standard intervention with prescribed energy-restricted diet and PA, and group intervention sessions.<br><br>IG2: Standard intervention enhanced with additional strategies at the initiation of intervention (ADOPT)<br><br>IG3: Standard intervention enhanced with additional strategies at predetermined times over the intervention period (MAINTAIN)	Goal setting (behaviour)<br>Problem solving (G2; G3)<br>Feedback on behaviour<br>Self-monitoring of behaviour<br>Instruction on how to perform the behaviour (IG2; IG3)<br>Demonstration of behaviour (IG2; IG3)<br>Behavioural practice/rehearsal (G2; G3)	Face-to-face	18 months	Self-reported PA - Energy expenditure in PA and bouts of PA performed over the previous week (Questionnaire developed for the Harvard Alumni Study).<br><br>At baseline, 6, 12, and 18 months.	Overall, there was a non-significant time effect for change in PA energy expenditure. No significant group–time interaction.

Authors, Year	Study Design	Sample	Intervention				Outcomes, Instruments & Time points	Physical Activity Changes
			Description	Theory & Strategies	Mode of Delivery	Length + Follow-up		
Jamal, 2016	RCT (2 arms)	GSlim (IG): 97 participants (72.1% women); Age, 32.4 ± 4.8 yrs; BMI, 39.7 ± 9.2 kg/m^2<br><br>Comparison (CG): 97 participants (74.2% women); 32.4 ± 3.8 yrs; BMI 40.4 ± 9.5 kg/m^2	IG: Group-approach intervention based on self-monitoring for weight, dietary, and PA, problem solving, motivation, and specific weight loss goals (10 sessions)<br><br>CG: Standard care with individual counselling (1 hour) with a dietitian once every 12 weeks	Goal setting (behaviour) (IG)<br>Problem solving (IG)<br>Goal setting (outcome) (IG)<br>Feedback on behaviour (IG)<br>Self-monitoring of behaviour (IG)<br>Self-monitoring of outcomes of behaviour (IG)<br>Feedback on outcomes of behaviour (IG)<br>Instruction on how to perform the behaviour (IG)<br>Demonstration of behaviour (IG)<br>Behavioural practice/rehearsal (IG)<br>Graded tasks (IG)<br>Reduce negative emotions (IG)<br>Framing/reframing (IG)	Face-to-face	24 weeks + 12 weeks follow up	Self-reported PA (International Physical Activity Questionnaire)<br><br>At baseline, 24 and 36 weeks	No significant change in PA for IG at 24 weeks. Moderate PA reduced significantly in the IG compared to the CG during the follow up period (from 24 to 36 weeks)
Johnson, 2008	RCT (2 arms)	IG1: 627 participants (45.6% women); Age, 45.3 yrs; BMI, 30.6 kg/m^2<br><br>CG: 649 participants (49.2% women); Age, 45.4 yrs; BMI, 30.9 kg/m^2	IG: Participants received four computer-generated tailored reports for multiple behaviour based on assessments (baseline, 3, 6 and 9 months). Reports were tailored on TTM constructs (stage of change, decisional balance, self-efficacy, and process of change)<br><br>CG: No treatment	Transtheoretical Model<br><br>Goal setting (IG)<br>Feedback on behaviour (IG)<br>Social comparison (IG)	Face-to-face	12 + 12 months	Self-reported PA (Godin Leisure Time Exercise Questionnaire)<br><br>At baseline, 3, 6, 9 12, and 24 months	Among those in pre-action stages for exercise at baseline, there was a significant group effect beginning at 6 months that was maintained over time. More participants from the IG progressed to action/maintenance phase than from the CG.
Kalter-Leibovici et al., 2010	RCT (2 arms)	Intensive (IG): 100 women; Age, 43.8 ± 5.6 yr; BMI, 34.0 ± 3.1 kg/m2<br><br>Moderate (CG): 101 women; Age, 44.0 ± 5.9 yrs; BMI, 33.8 ± 2.8 kg/m2	IG: 1 individual + 1 group session with dietitian plus 2 physical activity group sessions per month (strength and aerobic exercise; instructions for home exercise)<br><br>CG: 3 individual sessions (0, after 6 and 12 months) + 2 group sessions with dietitian (1st month); no guided physical activity	Self-monitoring of behaviour (IG)<br>Instruction on how to perform the behaviour (IG)<br>Behavioural practice/rehearsal (IG)	Face-to-face	12-months	Self-reported PA to assess % meeting physical activity recommendations – 150 minutes/week (Validated Hebrew Questionnaire)<br><br>At baseline, 6 and 12 months	Changes at 6 months:<br>Intensive: 30% over 150 minutes/week vs. 5% at baseline<br>Moderate: no change.<br><br>Changes at 12 months:<br>Intensive: 26% over 150 minutes/week vs. 5% at baseline<br>Moderate: 13.9% over 150 minutes/week vs. 6.9% at baseline

Authors, Year	Study Design	Sample	Intervention				Outcomes, Instruments & Time points	Physical Activity Changes
			Description	Theory & Strategies	Mode of Delivery	Length + Follow-up		
Latner et al., 2013	RCT (2 arms)	Total: 90 participants (64% women); Age, 49.7 ± 12.3 yrs<br><br>Standard Care (IG1): 38 participants; BMI, 36.1 ± 7.8 kg/m^2<br><br>Continued Care (IG2): 52 participants; BMI, 35.6 ± 8.1 kg/m^2	IG1/IG2: Both groups received 6-month weight loss lifestyle intervention, with 20 group sessions, focused on long-term healthy eating and PA, plus manual with additional behavioral strategies and skills.<br><br>IG2: Participants were further taught continuing care self-support strategies and instructed to continue meeting weekly on their own throughout the 18 months follow-up.	Goal setting (behaviour)<br>Behavioural contract (IG2)<br>Monitoring of behaviour by others without feedback (IG2)<br>Monitoring of outcomes of behaviour by others without feedback (IG2)<br>Social support (unspecified) (IG2)<br>Social reward (IG2)	Face-to-Face	6 months + 18 months follow-up	Self-reported PA (International Physical Activity Questionnaire)<br><br>At baseline, 6, 12 and 24 months	There were significant main effects of time for post-treatment walking and for 6-month follow-up moderate physical activity, having baseline as reference.
Leermakers et al., 1999	RCT; 2 arms	Total: 67 participants (80% women); Age, 50.8 ± 11.1 yrs<br><br>Exercise-focused maintenance (IG1): 38 participants; BMI, 35.6 ± 8.1 kg/m^2<br><br>Weight-focused maintenance (IG2): 29 participants; 36.1 ± 7.8kg/m^2	All participants were instructed to walk 30 minutes per day, 5 days per week<br><br>IG1: Multifaceted intervention designed to sustain the maintenance of PA, 13 biweekly supervised group exercise sessions; intergroup competitions and prizes based on group exercise completion.<br><br>IG2: Therapist-led group discussions focused on weight loss maintenance (13 biweekly sessions). Participants determined issues addressed during sessions. Therapists led group problem solving of weight-related difficulties presented by participants. No supervised exercise sessions, and no incentives for exercise completion.	Goal setting (behaviour)<br>Problem solving (IG1)<br>Self-monitoring of behaviour<br>Behavioural practice/rehearsal (IG1)<br>Material reward (behaviour) (IG1)<br>Non-specific reward (IG1)<br>Social incentive (IG1)<br>Reward (outcome) (IG1)	Face-to-Face	6-month initial weight loss interventio n + 6-month weight maintenan ce interventio n + 6-month follow-up	Objective PA (participant's records; Caltrac Accelerometers)<br><br>Self-reported PA (physical activity diaries)<br><br>At baseline, 6, 12 and 18 months	During the extended therapy phase (i.e., months 7 to 12), participants in both conditions reported significant increases in their weekly amounts of walking and no significant between-group differences were found.<br><br>No significant changes in energy expenditure over time for either group, and no between-group differences at any of the assessments.

Authors, Year	Study Design	Sample	Intervention				Outcomes, Instruments & Time points	Physical Activity Changes
			Description	Theory & Strategies	Mode of Delivery	Length + Follow-up		
Lindstrom et al., 2003	RCT (2 arms)	Intervention (IG): 265 participants (66% women); Age, 55 ± 7 yrs; BMI, 31.4 ± 4.5 kg/m^2<br><br>Control (CG): 257 (68% women); Age, 55 ± 7 yrs; BMI, 31.1 ± 4.5 kg/m^2	IG: Dietary and exercise face-to-face individualized sessions. Supervised, gradual, tailored moderate resistance training circuit sessions plus an exercise competition was offered. Discussions focused on individual problems.<br><br>CG: General information about lifestyle and diabetes risk and print material were delivered. The messages were the same, but counseling was not individualized.	Goal setting (IG)<br>Self-monitoring of behaviour (IG)<br>Behavioural practice/rehearsal (IG)<br>Graded tasks (IG)<br>Non-specific reward (IG)<br>Social incentive (IG)	Face-to-Face	12 months + 2-year follow-up	Self-reported PA (validated Kuopio Ischaemic Heart Diseas Risk Factor Study Leisure-Time Physical Activity Questionnaire)<br><br>At baseline and at every annual visit.	The total LTPA did not change, but moderate-vigorous LTPA increased in the intervention group compared with the control group at years 1 and 3.
Nakade et al., 2012	RCT (2 arms)	Intervention (IG): 58 men; Age, 53.6 ± 6.7 yrs; BMI, 29.8 ± 2.3 kg/m^2 + 57 women; Age, 55.1 ± 6.4 yrs; BMI, 30.9 ± 3.0 kg/m^2<br><br>Control (CG): 55 men; Age, 53.7 ± 6.3 yrs; BMI, 30.5 ± 3.7 kg/m^2 + 56 women; Age, 54.2 ± 6.2 yrs; BMI, 31.1 ± 3.1 kg/m^2	IG: Individual counseling (30 minutes) and group sessions about effective exercise (20 minutes) provided by registered dietitians and exercise instructors at baseline and at 1, 3, 6 and 9 months. Exercise instructor taught participants effective exercises for weight loss and participants mimicked the motions. In the individual counseling sessions, participants discussed lifestyle habits (dietary and physical activities) that needed improvement and set monthly plans to modify them.<br><br>CG: no support	Goal setting (IG)<br>Action planning (IG)<br>Review behaviour goals (IG)<br>Feedback on behaviour (IG)<br>Self-monitoring of behaviour (IG)<br>Social support (unspecified) (IG)<br>Instruction on how to perform the behaviour (IG)<br>Demonstration of behaviour (IG)<br>Behavioural practice/rehearsal (IG)<br>Graded tasks (IG)<br>Social reward (IG)	Face-to-Face	12 months + 12-month follow-up	Objective PA - daily step counts (uniaxial accelerometer)<br><br>At baseline and 12 months for all, and at 24 months for the IG	The intervention group walked more after the 12 months, both genders, than at the beginning of the program, and significant group-by-time interactions were seen in both sexes. Group-by-time interactions at follow-up remained significant although the number of steps decreased in both genders.

Authors, Year	Study Design	Sample	Intervention				Outcomes, Instruments & Time points	Physical Activity Changes
			Description	Theory & Strategies	Mode of Delivery	Length + Follow-up		
Newton Jr., et al 2018	RCT (2 arms)	Intervention (IG): 68 participants (94% women); Age, 54.9 ± 10.7 yrs; BMI, 38.9 ± 6.7 kg/m^2<br>Control (CG): 29 participants (86% women); Age, 58.6 ± 8.7 yrs; BMI, 37.7 ± 5.6 kg/m^2	IG: Group sessions and behavioural change content via SMS text messages focused on healthy eating and PA.<br>CG: Delayed intervention with 3 SMS text messages with general info to promote weight loss	Goal setting (IG)<br>Problem solving (IG)<br>Self-monitoring of behaviour (IG)<br>Social support (unspecified) (IG)<br>Information about health consequences (IG)<br>Information about others approval (IG)<br>Social reward (IG)<br>Reduce negative emotions (IG)	Face-to-face and SMS	6 months	Self-reported PA (International Physical Activity Questionnaire)<br>At baseline and 6 months	No significant between-group differences were observed in MET-minutes/week.
Overgaard et al., 2018	RCT (2 arms)	SitLess (IG1): 23 participants (65% women); Age, 45.0 ± 11.5 yrs; BMI, 32.3 ± 4.1 kg/m^2<br>ExMore (IG2): 20 participants (70% women); Age, 46.1 ± 10.3 yrs; BMI, 34.4 ± 5.8 kg/m^2	IG1: This group was instructed to reduce sedentary behavior during the 4 weeks. A list of non-sedentary activities to replace sitting activities during time at home, work, leisure, or transport was presented and handed-out.<br>IG2: Instructed to increase MVPA to at least 30 minutes per day (using activity types of their own choosing).	Goal setting (behaviour) (IG2)<br>Self-monitoring of behaviour<br>Instruction on how to perform the behaviour (IG1)<br>Demonstration of behaviour (IG1)	Face-to-Face	4 weeks	Objective PA - time spent in MVPA (accelerometers).<br>At baseline and 4 weeks	IG2 increased MVPA, while IG1 did not.
Rapoport et al., 2000	RCT (2 arms)	M-CBT (IG1): 37 women; Age, 49 ± 10 yrs; BMI, 35.4 ± 6.3 kg/m^2<br>S-CBT (IG2): 38 women; Age, 46 ± 12 yrs; BMI, 35.3 ± 5.6 kg/m^2	Both treatment programs involved weekly, 2-hour sessions over a 10-week period.<br>IG1: Aim was lifestyle change. Participants were encouraged to start a walking program at an appropriate level and adding an extra 5 min per week. Additional forms of PA were explored using motivational interviewing.<br>IG2: Aim was to achieve weight loss through energy restriction. Sessions included education on healthy eating (energy restriction) and PA.	Problem solving (IG1)<br>Goal setting (outcome) (IG2)<br>Self-monitoring of behaviour<br>Self-monitoring of outcomes of behaviour (IG2)<br>Instruction on how to perform the behaviour (IG2)<br>Monitoring of emotional consequences (IG1)<br>Demonstration of behaviour (IG2)<br>Graded tasks (IG1)<br>Reduce negative emotions (IG1)	Face-to-Face	10-week + 12-month follow-up	Self-reported PA and energy expenditure (Questionnaire, adapted from Taylor et al., that assessed frequency, duration, and intensity, over the past month).<br>At baseline, end of treatment, and 6- and 12-month follow-ups.	There was a significant increase in reported physical activity, but no group by time interaction.

Authors, Year	Study Design	Sample	Intervention				Outcomes, Instruments & Time points	Physical Activity Changes
			Description	Theory & Strategies	Mode of Delivery	Length + Follow-up		
Schelling et al., 2009	RCT (2 arms)	Motivational intervention (IG1): 18 participants (67% women); Age, 51.9 ± 7.2 yrs; BMI, 32.8 ± 3.1 kg/m^2<br><br>Relaxation intervention (IG2): 20 participants (80% women); Age, 45.2 ± 11.2 yrs; BMI, 32.4 ± 3.5 kg/m^2	IG1: Motivation session aimed at establishing solid knowledge about possible barriers to increase PA and to prepare participants to deal with difficulties<br><br>IG2: Relaxation session using the progressive muscle relaxation technique	Problem solving (IG1)<br>Instruction on how to perform the behaviour (IG1)<br>Information about health consequences (IG1)<br>Information about emotional consequences (IG1)<br>Demonstration of behaviour (IG1)<br>Behavioural practice/rehearsal<br>Graded tasks<br>Pros and cons (IG1)	Face-to-Face	8 weeks + 6-month follow-up	Self-reported PA (weekly exercise diaries)<br><br>During the aerobic program and at 3- and 6-month follow-ups	In IG1, the minutes spent on PA first increased over time and then leveled off, whereas steady decreases were observed in IG2.
Share et al., 2015	RCT (2 arms)	Intervention (IG): 28 women; Aged 18-30 yr; BMI, 32.2 ± 5.9 kg/m^2<br><br>Control (CG): 17 women; Aged 18-30 yr; BMI, 31.4 ± 6.6 kg/m^2	IG: 12-week lifestyle intervention with 3 main components: (1) PA (2) nutrition education, and (3) cognitive behavioral therapy. Participants undertaking the intervention completed two supervised exercise sessions.<br><br>CG: Instructed to continue existing lifestyle choices, and after 12 weeks were invited to complete the lifestyle intervention	Self-Determination Theory<br><br>Goal setting (behaviour) (IG)<br>Problem solving (IG)<br>Social support (unspecified) (IG)<br>Social support (emotional) (IG)<br>Behavioural practice/rehearsal (IG)	Face-to-Face	12 weeks	Self-reported PA (7-day PA recall)<br><br>At baseline and 12 weeks	Physical activity increased in the intervention group and it did not change in the control group.

Authors, Year	Study Design	Sample	Intervention				Outcomes, Instruments & Time points	Physical Activity Changes
			Description	Theory & Strategies	Mode of Delivery	Length + Follow-up		
Silva et al., 2011	RCT (2 arms)	221 women; Age, 37.6 ± 7.0 yrs; BMI, 31.6 ± 4.1 kg/m^2	IG: Group attended 30 sessions, targeted at increasing PA and energy expenditure, adopting a diet consistent with a moderate energy deficit, and integrating exercise and eating patterns that would support weight maintenance. Special focus was on increasing autonomous regulation toward exercise and weight control.<br><br>CG: 29-session general health education curriculum based on several educational courses covering various topics (e.g., preventive nutrition, stress management, self-care, and effective communication skills).	Self-Determination Theory<br><br>Goal setting (behaviour) (IG)<br>Problem solving (IG)<br>Action planning (IG)<br>Discrepancy between current behaviour and goal (IG)<br>Self-monitoring of behaviour (IG)<br>Social support (unspecified) (IG)<br>Instruction on how to perform the behaviour (IG)<br>Information about antecedents (IG)<br>Monitoring of emotional consequences (IG)<br>Demonstration of behaviour (IG)<br>Social comparison (IG)<br>Behavioural practice/rehearsal (IG)<br>Pros and cons (IG)<br>Social reward (IG)<br>Self-reward (IG)<br>Framing/reframing (IG)	Face-to-Face	12 months + 24 months follow-up	Self-reported MVPA (7-day physical activity recall).<br><br>At baseline, 12, 24, 36 months	There was a significant difference in 24-month MVPA between groups, favoring the IG.
Silva et al., 2010	RCT (2 arms)	Intervention (IG): 123 women; Age, 38.1 ± 7 yrs; BMI, 31.7 ± 11.9 kg/m^2<br><br>Control (CG): 116 women; Age, 37.1 ± 7; BMI, 31.3 ± 4 kg/m^2	IG: Group attended 30 sessions, targeted at increasing PA and energy expenditure, adopting a diet consistent with a moderate energy deficit, and integrating exercise and eating patterns that would support weight maintenance. Special focus on increasing competence and autonomous regulation toward exercise and weight control.<br><br>CG: 29-session general health education curriculum based on several educational courses covering various topics (e.g., preventive nutrition, stress management, self-care, and effective communication skills).	Self-Determination Theory<br><br>Goal setting (behaviour) (IG)<br>Problem solving (IG)<br>Action planning (IG)<br>Discrepancy between current behaviour and goal (IG)<br>Self-monitoring of behaviour (IG)<br>Social support (unspecified) (IG)<br>Instruction on how to perform the behaviour (IG)<br>Information about antecedents (IG)<br>Monitoring of emotional consequences (IG)<br>Demonstration of behaviour (IG)<br>Social comparison (IG)<br>Behavioural practice/rehearsal (IG)<br>Pros and cons (IG)<br>Social reward (IG)<br>Self-reward (IG)<br>Framing/reframing (IG)	Face-to-Face	12 months	Self-reported MVPA (7-day physical activity recall).<br><br>Lifestyle PA (Activity Choice Index).<br><br>At baseline and 12 months.	Both types of PA were significantly different at 12 months favouring the IG.

Authors, Year	Study Design	Sample	Intervention				Outcomes, Instruments & Time points	Physical Activity Changes
			Description	Theory & Strategies	Mode of Delivery	Length + Follow-up		
Simpson et al., 2015	RCT (3 arms)	Intensive (IG1): 54 participants (83.3% women); 59% aged 30-59 yr; BMI, 34.4 ± 6.2 kg/m^2<br><br>Less intensive (IG2): 54 participants (83.3% women); 61% aged 30-59 yr; BMI, 34.8 ± 6.2 kg/m^2<br><br>Control (CG): 58 participants (84.5% women); 62.5% aged 30-59 yr; BMI, 33.3 ± 5.2 kg/m^2	IG1: Six 1-hour face-to-face sessions during the first 3 months followed by nine 20-min telephone sessions.<br><br>IG2: Two 1-hour face-to-face sessions during the first month followed by two 20-min telephone sessions.<br><br>Control: Leaflet advising on healthy eating and lifestyle along with usual care.	Self-Determination Theory, Social Cognitive Theory, Self-monitoring, Model of Action Phases, Social Support<br><br>Goal setting (behaviour) (IG1; IG2)<br>Problem solving (IG1; IG2)<br>Goal setting (outcome) (IG1; IG2)<br>Action planning (IG1; IG2)<br>Discrepancy between current behaviour and goal (IG1; IG2)<br>Feedback on behaviour (IG1; IG2)<br>Self-monitoring of behaviour (IG1; IG2)<br>Self-monitoring of outcome of behaviour (IG1; IG2)<br>Social support (unspecified) (IG1; IG2)<br>Credible source (IG1; IG2)<br>Non-specific reward (IG1; IG2)	Face-to-Face (+ telephone)	12 months	Self-reported PA (International Physical Activity Questionnaire)<br><br>At baseline and 12 months.	There was no evidence of any impact of treatment group on PA, but IG1 and IG2 had a greater score than that the CG.
Steptoe et al., 2000	RCT (2 arms)	Total: 699 participants (54% women); Age, 49.1 ± 11.2 yrs; BMI, 29.2 ± 2.7 kg/m^2	IG: Behaviorally oriented counseling with methods varying with stage of readiness. No supervised exercise was offered. Education about exercise and health.<br><br>CG: Usual health promotion practices to encourage increased physical activity in sedentary patients.	Stage of Change Model<br><br>Goal setting (behaviour) (IG)<br>Problem solving (IG)<br>Self-monitoring of behaviour (IG)<br>Instruction on how to perform the behaviour (IG)<br>Information about health consequences (IG)<br>Demonstration of behaviour (IG)<br>Non-specific incentive (IG)<br>Framing/reframing (IG)	Face-to-Face	12 months	Self-reported PA (questions from the UK National Fitness Survey - number and duration of 15 different activities)<br><br>At baseline, 4 and 12 months.	Greater increases in physical activity at 4 and 12 months vs. controls

Authors, Year	Study Design	Sample	Intervention				Outcomes, Instruments & Time points	Physical Activity Changes
			Description	Theory & Strategies	Mode of Delivery	Length + Follow-up		
Thiabpho et al., 2018	RCT (2 arms)	Intervention (IG): 30 women; Age, 43.8 ± 4.8 yrs; BMI, 31.9 ± 3.7 kg/m^2<br><br>Control (CG): 30 women; Age, 43.0 ± 0.7 yrs; BMI, 32.7 ± 3.9 kg/m^2	IG: Consisted of 12 sessions, once a week for the first eight weeks and then every two weeks until the 16th week. There were three main components of the intervention conducted including (1) tailored nutrition counseling using motivational interviewing, (2) health education aimed at increasing the severity, susceptibility and threats regarding obesity and health, understanding benefits and barriers relating to weight loss, increasing individual's ability regarding diet and exercise and stimulating cues to lose weight, and (3) an exercise training session to increase individual's ability in performing exercise.	Health belief model<br><br>Goal setting (behaviour) (IG)<br>Goal setting (outcome) (IG)<br>Self-monitoring of behaviour (IG)<br>Self-monitoring of outcomes of behaviour (IG)<br>Social support (unspecified) (IG)<br>Information about health consequences (IG)<br>Demonstration of behaviour (IG)<br>Social comparison (IG)<br>Behavioural practice/rehearsal (IG)<br>Pros and cons (IG)<br>Social reward (IG)	Face-to Face	16 weeks	Self-reported PA. Each participant kept a physical activity record log with a specific time of 1 week. The average physical activity in METs was estimated.	At the program completion, the mean score of physical activity for the intervention group showed statistically greater improvement than the control group.
Volger et al., 2013	RCT (3 arms)	Brief Lifestyle Counseling (IG1): 131 participants; Age, 52 ± 12.2 yrs; BMI, 38.5 ± 4.6 kg/m^2<br>Enhanced Brief Lifestyle Counselling (IG2): Age, 51.0 ± 10.1 yrs; BMI, 37.8 ± 4.7 kg/m^2<br>Usual care (CG): 130 participants; Age, 51.7 ± 12.1 yrs; BMI, 39 ± 4.8 kg/m^2	IG1: Participants met monthly with an auxiliary health-care provider who instructed them regarding diet and activity modification during 10- to 15-min sessions. These lessons presented a variety of behavioural strategies.<br><br>IG2: Participants also chose to receive either meal replacements or weight loss medication<br><br>Usual Care (CG)	Goal setting (behaviour)<br>Problem solving (IG1; IG2)<br>Review behaviour goals (IG1; IG2)<br>Self-monitoring of behaviour (IG1; IG2)<br>Instruction on how to perform the behaviour (IG1; IG2)<br>Demonstration of the behaviour<br>Graded tasks (IG1; IG2)<br>Reduce negative emotions (IG1; IG2)<br>Framing/reframing (IG1; IG2)	Face-to-face	6 months + 18-month follow-up	Self-reported PA (Paffenbarger Physical Activity Survey)<br><br>Objective PA (Pedometer W4lQ6622)<br><br>At baseline, 6 and 24 months	Energy expenditure from MVPA decreased in CG at month 6, whereas it increased in both IGs. Increases in energy expenditure from MVPA were significantly greater in IG2 compared with the other two groups, and IG1 was superior to CG.

Note. IG, Intervention group; CG, Control group

Table S3. Quality of included original controlled studies.

References	Criteria														Total "Yes"	Total "No"	Total "other"	Quality rating
	1	2	3	4	5	6	7	8	9	10	11	12	13	14				
Adams_2017	Y	Y	Y	N	Y	Y	Y	Y	Y	Y	Y	Y	Y	Y	13	1	0	GOOD
Akers_2012	Y	NR	CD	NR	NR	N	Y	Y	Y	N	N	Y	Y	CD	6	3	5	POOR
Ash_2006	Y	Y	Y	NR	NR	Y	N	N	N	NR	Y	NR	Y	Y	7	3	4	FAIR
Berli_2018	Y	Y	Y	NR	NR	Y	Y	Y	Y	Y	Y	N	Y	Y	11	1	2	GOOD
Biddle_2015	Y	Y	Y	N	NR	NR	N	N	N	Y	Y	N	Y	Y	7	5	2	FAIR
Block_2016	Y	Y	Y	Y	Y	Y	Y	Y	Y	Y	Y	Y	Y	Y	14	0	0	GOOD
Boiché_2018	N	N	N	NR	NR	Y	N	CD	CD	N	Y	NR	Y	CD	3	5	6	POOR
Carr_2008	Y	CD	CD	NR	NR	Y	Y	Y	N	NR	Y	Y	Y	Y	8	1	5	GOOD
Carr_2013	Y	Y	Y	Y	Y	Y	Y	N	Y	CD	Y	Y	Y	Y	12	1	1	GOOD
Cohen_2017	N	N	N	NR	NR	N	NR	NR	CD	NR	Y	NR	Y	CD	2	4	8	POOR
Collins_2012	Y	Y	Y	Y	Y	Y	Y	N	Y	CD	Y	Y	Y	Y	12	1	1	GOOD
Conroy_2015	Y	Y	Y	NR	NR	Y	N	N	Y	Y	Y	NR	Y	Y	9	2	3	FAIR
Conroy_2011	Y	Y	CD	NR	NR	Y	Y	Y	Y	Y	Y	CD	Y	Y	10	0	4	GOOD
Eaton_2016	Y	Y	Y	N	Y	Y	N	Y	Y	Y	Y	Y	Y	Y	12	2	0	FAIR
Gill_2019	Y	Y	Y	N	NR	Y	N	Y	Y	CD	Y	Y	Y	Y	10	2	2	FAIR
Greaney_2017	Y	Y	Y	Y	NR	Y	Y	Y	Y	CD	Y	Y	Y	N	11	1	2	FAIR
Gohner_2012	N	N	N	NR	NR	N	N	Y	N	Y	Y	N	Y	CD	4	7	3	POOR
Hales_2016	Y	Y	Y	N	N	Y	Y	Y	Y	Y	Y	CD	Y	Y	11	2	1	GOOD
Hochsmann_2019	Y	Y	Y	Y	Y	Y	Y	Y	Y	Y	CD	Y	Y	Y	13	0	1	FAIR
Hurkmans_2018	Y	Y	Y	CD	Y	Y	N	Y	Y	CD	Y	CD	Y	Y	10	1	3	FAIR
Hutchesson_2018	Y	Y	Y	NR	Y	Y	N	Y	Y	Y	Y	N	Y	Y	11	2	1	FAIR
Jakicic_2012	Y	Y	Y	CD	Y	Y	N	Y	CD	CD	Y	Y	Y	Y	10	1	3	FAIR
Jakicic_2015	Y	Y	Y	N	N	Y	Y	Y	Y	CD	CD	N	Y	Y	9	3	2	FAIR
Jamal_2016	Y	Y	Y	Y	NR	Y	Y	Y	Y	CD	Y	Y	Y	Y	12	0	2	GOOD
Johnson_2008	Y	CD	CD	CD	CD	CD	N	Y	N	Y	Y	NR	Y	CD	5	2	7	POOR
Kalter-Leibivici_2010	Y	Y	Y	N	Y	Y	Y	Y	Y	NR	N	Y	Y	Y	11	2	1	FAIR
Kurtzman_2018	Y	NR	Y	N	Y	Y	Y	Y	CD	Y	Y	Y	Y	Y	11	1	2	GOOD
Laing_2014	Y	Y	Y	Y	Y	NR	N	Y	N	N	N	Y	Y	NR	8	4	2	POOR
Latner_2013	Y	Y	Y	NR	NR	NR	Y	Y	N	CD	N	Y	Y	Y	8	2	4	FAIR
Leermarkers_1999	Y	CD	CD	NR	NR	Y	Y	Y	N	CD	Y	N	Y	Y	7	2	5	GOOD
Lindstrom_2003	Y	Y	CD	NR	NR	Y	Y	Y	CD	Y	N	Y	Y	N	8	2	4	POOR

McConnon_2007	Y	Y	Y	N	N	Y	N	N	CD	CD	N	Y	Y	Y	7	5	2	POOR
Mensorio_2019	Y	Y	Y	NR	NA	Y	N	N	CD	CD	N	Y	Y	Y	7	3	4	POOR
Morgan_2011	Y	Y	Y	N	NR	Y	Y	Y	N	CD	N	Y	Y	Y	9	3	2	FAIR
Nakade_2012	Y	NR	Y	NR	NR	Y	Y	Y	NR	Y	Y	N	Y	N	7	2	4	FAIR
Napolitano_2013	Y	NR	Y	NR	NA	Y	Y	Y	N	Y	N	N	Y	N	7	4	3	POOR
Newton_2018	Y	Y	Y	N	CD	Y	N	CD	N	CD	Y	N	Y	Y	7	4	3	FAIR
Overgaard_2018	Y	Y	Y	N	NR	Y	Y	Y	NR	NR	Y	Y	Y	N	9	2	3	FAIR
Patrick_2011	Y	Y	Y	N	Y	Y	N	Y	Y	NR	N	Y	Y	Y	10	3	1	POOR
Rapoport_2000	Y	Y	Y	N	NR	N	Y	Y	Y	NR	N	N	Y	N	7	5	2	POOR
Richardson_2010	Y	Y	Y	NR	NR	N	N	Y	Y	NR	Y	Y	Y	Y	9	2	3	FAIR
Schelling_2009	Y	Y	Y	NR	NR	CD	N	Y	NR	NR	N	N	Y	Y	6	3	5	POOR
Shapiro_2012	Y	Y	Y	N	N	Y	N	Y	N	NR	Y	Y	Y	Y	9	4	1	FAIR
Share_2015	Y	N	Y	N	Y	Y	N	Y	Y	NR	N	N	Y	N	7	6	1	POOR
Silva_2011	Y	Y	Y	NR	NR	Y	Y	Y	NR	Y	N	Y	Y	Y	10	1	3	FAIR
Silva_2010	Y	Y	Y	NR	NR	Y	Y	Y	NR	Y	N	Y	Y	Y	10	1	3	FAIR
Simpson_2015	Y	Y	Y	N	N	Y	Y	Y	Y	NR	N	Y	Y	Y	10	3	1	FAIR
Sniehotta_2019	Y	Y	Y	N	Y	Y	Y	Y	NR	Y	Y	Y	Y	Y	12	1	1	GOOD
Steinberg_2013	Y	NR	NR	NR	NR	N	Y	Y	NR	NR	N	NR	Y	Y	5	2	7	FAIR
Stephens_2017	Y	NR	NR	NR	NR	Y	Y	Y	N	Y	N	Y	Y	N	7	3	4	POOR
Steptoe_1999	Y	NR	NR	NR	NR	Y	N	Y	NR	NR	N	Y	Y	N	5	3	6	POOR
Tate_2003	Y	Y	Y	NR	NR	Y	Y	Y	CD	NR	N	N	Y	Y	8	2	4	FAIR
Tate_2006	Y	Y	Y	NR	NR	Y	Y	Y	CD	NR	N	Y	Y	Y	9	1	4	FAIR
Thiabpho_2018	Y	NR	NR	NR	NR	Y	Y	Y	CD	Y	N	Y	Y	N	7	2	4	POOR
Turner-McGrievy_2011	Y	Y	Y	N	N	Y	Y	Y	Y	Y	Y	Y	Y	Y	12	2	0	GOOD
Turner-McGrievy_2009	Y	CD	CD	Y	CD	Y	Y	Y	Y	CD	Y	NR	Y	Y	9	0	5	GOOD
van Genugten_2012	Y	Y	Y	CD	CD	Y	N	N	N	CD	Y	Y	Y	N	7	4	3	POOR
van Wier_2009	Y	Y	Y	N	N	CD	N	Y	N	CD	Y	N	Y	CD	6	5	3	POOR
Volger_2013	Y	Y	Y	CD	CD	Y	N	Y	N	CD	Y	Y	Y	CD	8	2	4	POOR
Wang_2015	Y	Y	Y	N	CD	N	Y	Y	Y	CD	Y	Y	N	Y	9	3	2	GOOD
Watson_2012	Y	Y	Y	N	N	Y	Y	Y	Y	CD	CD	N	Y	CD	8	3	3	POOR
Watson_2015	Y	Y	Y	N	Y	Y	N	Y	Y	Y	Y	N	Y	Y	11	3	0	FAIR
West_2016	Y	CD	CD	CD	CD	Y	Y	Y	Y	CD	N	CD	Y	Y	7	1	6	FAIR

Notes. Criteria controlled trials: (1) Randomized study; (2) Adequate randomization method; (3) Treatment allocation concealment; (4) Blinding treatment assignment; (5) Blinding outcome assessors; (6) Similar baseline characteristics; (7) Drop-out rate <20%; (8) Differential drop-out rate between groups <15%; (9) High adherence; (10) Similar background treatments; (11) Valid and reliable outcome measures; (12) Sample size justification; (13) Pre-specified outcomes/subgroups; (14) All randomized participants analysed (ITT analysis).
